# Wide area spray of bacterial larvicide, *Bacillus thuringiensis israelensis* strain AM65-52, integrated in the national vector control program impacts dengue transmission in an urban township in Sibu district, Sarawak, Malaysia

**DOI:** 10.1371/journal.pone.0230910

**Published:** 2020-04-01

**Authors:** Rezal Bohari, Chong Jin Hin, Asmad Matusop, Muhamad Rais Abdullah, Teoh Guat Ney, Seleena Benjamin, Lee Han Lim

**Affiliations:** 1 Sibu Divisional Health Office, Sibu, Sarawak, Malaysia; 2 Sarawak State Health Department, Kuching, Sarawak, Malaysia; 3 Medical Entomology Unit, Institute For Medical Research, Kuala Lumpur, Malaysia; 4 Valent BioSciences LLC, Malaysia; Institut Pasteur, FRANCE

## Abstract

Several sites, Z-7L, Z-5 and Z-14, in Sibu district, Sarawak, Malaysia, experienced intense dengue transmission in 2014 that continued into 2015. A pilot study with *Bacillus thuringiensis israelensis* (Bti) to control *Aedes aegypti* (L.) and *Ae*. *albopictus* (Skuse) was evaluated in Z-7L, a densely populated site of 12 ha. Bti treatments were conducted weekly from epidemiology week (EW) 24/2015 for 4 weeks, followed by fortnight treatments for 2 months, in addition to the routine control activities. Bti was directly introduced into potable containers and the outdoor artificial and natural containers were treated via a wide area spray application method using a backpack mister. *Aedes* indices significantly reduced during the treatment and post treatment phases, compared to the control site, Z-5 (p<0.05). A 51 fold reduction in the incidence rate per 100,000 population (IR) was observed, with one case in 25 weeks (EW 29–52). In Z-5 and Z-14, control sites, a 6 fold reduction in the IR was observed from EW 29–52. However, almost every week there were dengue cases in Z-14 and until EW 44 in Z-5. In 2016, dengue cases resurfaced in Z-7L from EW 4. Intensive routine control activities were conducted, but the IR continued to escalate. The wide area Bti spray misting of the outdoor containers was then included from EW 27 on fortnight intervals. A 6 fold reduction in IR was observed in the Bti treatment phase (EW 32–52) with no successive weekly cases after EW 37. However, in the control sites, there were dengue cases throughout the year from EW 1–52, particularly in Z-14. We feel that the wide area Bti spray application method is an integral component in the control program, in conjunction with other control measures carried out, to suppress the vector population in outdoor cryptic containers and to interrupt the disease transmission.

## Introduction

Dengue is one of the serious and major public health mosquito borne diseases throughout tropical and sub-tropical regions of the world, including Malaysia. In Malaysia, since the year 2000, the number of dengue cases and number of deaths have increased, on average by 14% and 8% per year, respectively [1]. There is simultaneous circulation of all four dengue virus serotypes in Malaysia, with a predominant virus serotype changing from year to year and in between the states in Malaysia. Changes in the circulating dengue virus serotype in the community have led to an increase of dengue cases and deaths [2].

In Malaysia, the two dengue vectors are *Aedes aegypti* (L.) and *Ae*. *albopictus* (Skuse) [3]. They breed indoors and outdoors, with *Ae*. *aegypti* being a dominant indoor breeder and *Ae*. *albopictus* as the dominant outdoor breeder and they have an ability to colonize in varied type of artificial and natural containers [4–6].

Effective vector control is considered an important tool in the prevention and control of dengue virus transmission [7]. Vector control targets to shorten the longevity of immature and adult mosquitoes, with the objective of interrupting the transmission cycle. The current practice of national vector control program in Malaysia includes application of insecticides, from the class of pyrethroids (PY) and organophosphates (OP), through space spraying application via cold (ultra-low volume, ULV) or thermal fogging [8]. Chemical larvicide such as temephos, an OP compound, is in use for the control of mosquito larvae in potable water since the first Malaysian dengue fever outbreak in 1973 [9]. Although chemical insecticides were successful in controlling mosquitoes in the past decades, the development of resistance in the larval and adult mosquito population has posed a great challenge in the recent time for dengue control management in Malaysia [10–14]. There is widespread resistance in several States in West Malaysia particularly against temephos, adulticides PY and OP in the *Ae*. *aegypti* population [10–12]. In Sarawak, a State from East Malaysia, the temephos resistance for both *Ae*. *aegypti* and *Ae*. *albopictus* larvae is documented [13], together with the resistance of the *Ae*. *albopictus* adult population to a range of insecticides from the 4 major insecticide classes (PY, OP, organochlorine and carbamates) [14].

Sibu District in Sarawak State, East Malaysia has been known to be dengue prone and intermittent outbreaks have been reported since the 1980’s. Since early 2014, Sibu District has been plagued with continuous episodes of dengue outbreaks. During the outbreaks, adulticiding with PY and OP was intensively conducted once every 2–3 days for a stretch of 10 days. Larviciding was also conducted with temephos and the *Bacillus thuringiensis israelensis* (Bti) aqueous formulation (VectoBac 12AS). The larvicides were applied using the hand pump or compression sprayers in the immediate surrounding of the dengue case homes. The search and destroy activities of all potential larval habitats were also included as an outbreak control measure. Despite intensive control measures there were persistent dengue episodes.

This paper reports on an operational pilot study conducted in 2015 in an area with high density of dengue within Zone 7 of Sibu township, site Z7-L. In this study, a bacterial larvicide, VectoBac WG, a water dispersible granule Bti formulation was applied and evaluated. VectoBac WG is a registered product for mosquito control with the Pesticide Board of Malaysia under the Pesticides Act 1974 [15]. Product is registered for direct application into potable-storage water containers and for wide area treatment of larval habitats through spray application. The National Dengue Strategic Plan of the Ministry of Health (MOH) for dengue prevention and control recommends the use of Bti in the integrated vector management (IVM) program during an outbreak or as a preventive control measure when the vector population exceeds the threshold values. This plan is revised and updated once every 5 years [16]. The 2015 study was conducted by the Sibu Divisional Health Office, and jointly supervised and supported by the Sarawak State Health Department, the Institute for Medical Research, Malaysia and Valent BioSciences. The impact of larviciding with VectoBac WG on dengue vector density and dengue transmission in site Z7-L is reported here together with data from the control sites, Z-5 and Z-14.

This paper also reports the integration of wide area spray application of VectoBac WG into routine operational dengue control program activities in 2016 and its impact on dengue transmission in site Z7-L. The impact is compared with control sites, Z-5 and Z-14. The 2016 activities were coordinated by the Sarawak State Health Department and the activities on the ground were executed by the Sibu Divisional Health Office.

## Materials and methods

### Pilot operational field efficacy study in the year 2015

The pilot operational field study was undertaken from May, epidemiological week (EW) 19 to October EW 41/2015. It was divided into 3 phases: pre-treatment phase, EW 19–23; Bti-treatment phase, EW 24–35; and post-Bti treatment phase, EW 36–41.

The Sarawak State Health Department reviewed and consented to the study procedure. The community leaders from the study sites attended the weekly dengue outbreak committee meetings which were chaired by the Sibu District Officer. At these meetings the study procedure was presented, and the community leaders gave the consent for the direct and wide area spray applications of VectoBac WG. During the study period, updates on the progress of the study were periodically given to the community leaders in the same meetings.

#### Description of the study site

Sarawak State is in the island of Borneo, and the state has 12 administrative divisions. Sibu Division is located at the central region of Sarawak with 3 districts, Sibu, Selangau and Kanowit. Since 2014, Sibu a low-lying riverine town had outbreak levels of dengue infection. By EW 8/2015, 62.4% of the total dengue cases in the state of Sarawak was reported in Sibu District [17].

Sibu District covers an area of 2,229.8 km^2^, with 2 local government councils, the Sibu Municipal Council (SMC) and Sibu Rural District Council (SRDC). SMC is further divided into 31 operational zones. For the pilot study we focused in Zone 5 (Z-5), Zone 7 (Z-7) and Zone 14 (Z-14). The zones were chosen based on the history of disease burden and frequent occurrence of dengue.

**Fig 1:**
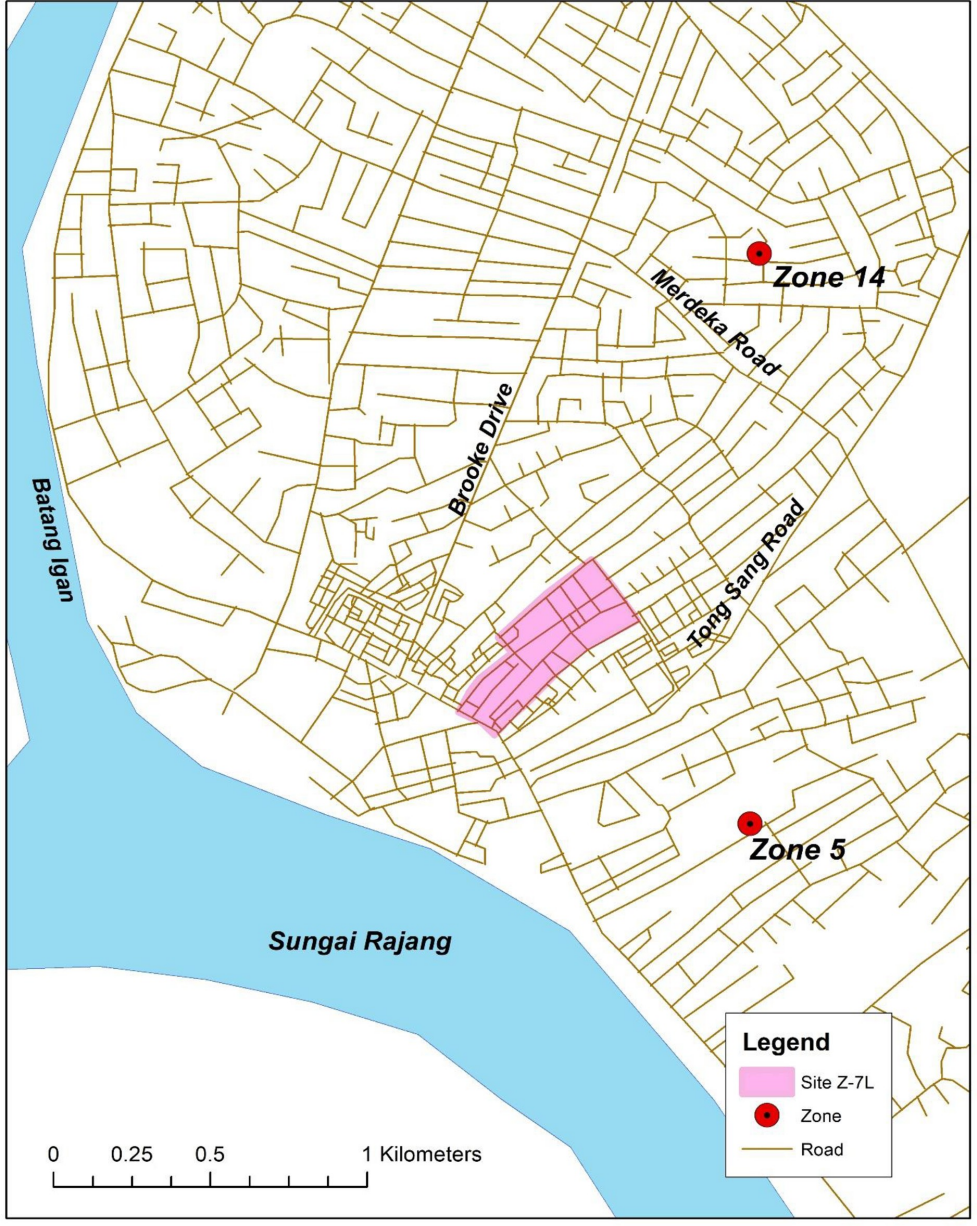
The shaded ■ area, site Z-7L of 12 ha, demarcated for wide area Bti spray treatment. A low lying riverine site, 500 meters from Rajang River. In Zone 5 and in Zone 14 the wide area Bti spray treatments were not conducted.

A 12 ha site in Zone 7 was demarcated for wide area spray Bti treatment and it was denoted as Z-7L (2° 17' 34.3602" N; 111° 49' 58.677" E) (Fig 1). The control sites were Zone 5 (Z-5) (2° 17' 4.4514"N; 111° 50' 5.0994" E) and Zone 14 (Z-14) (2° 18' 07.83" N; 111° 50' 17.53" E) where there was no Bti treatment activity. The 3 designated sites had similar housing structure amid dense vegetation and poor drainage with trash strewn in and outside the drains. The distance between the sites was approximately 0.4–0.8 km, separated by dual-carriageway roads, which served as a buffer zone to avoid inter-movement and crossing over of target vectors. All 3 zones are highly populated residential areas with brick or wooden houses and a commercial block. The Health Department and the Municipal Council carried out adulticiding in all 3 zones with PY and OP when dengue cases were reported.

#### Entomological surveillance

Entomological surveillance was conducted in Z-7L and in Z-5. Surveillance was conducted by the Entomology Team from Sibu Divisional Health Office for a continuous 23 weeks (EW 19–41) covering pretreatment, treatment and post treatment phases. The surveillance was conducted following the guideline as described under the Destruction of Disease-Bearing Insects Act 1975 (DDBIA 1975) under the Laws of Malaysia [18]. Following the DDBIA 1975 guideline together with the oral consent of premise owners allowed the health staff to examine premises for larval breeding and conduct control measures to eliminate the breeding.

The entomological surveillance consisted of larvae-pupae surveillance and ovitrap surveillance.

*Larvae and pupae surveillance*. Larvae and pupae surveillance were conducted weekly from EW 19–41. All indoor and outdoor containers holding water were inspected in 15 houses per study site per surveillance. All larvae-pupae were removed from the containers and transferred to the entomology laboratory at the Lanang Health Clinic, Sibu for enumeration and identification. The early instar larvae (L1/L2) were reared to L3/L4 for more accurate identification.

*Ovitrap surveillance*. The population of adult female gravid *Aedes* spp mosquitoes was measured by ovitrap surveillance [19]. This surveillance was conducted once in 2 weeks from EW 19–41. A total of 30 traps per study site were placed and were retrieved 5 days later within the same epidemiology week. The ovitraps were then analyzed at the entomology laboratory. The contents of the ovitrap were transferred into individual plastic containers for another 5–7 days for the eggs to hatch. The larvae were identified and ovitrap index (OI) was determined.

The following entomological indices were determined:

- **Aedes Index (AI):** Percent houses infested with *Ae*. *aegypti* or/and *Ae*. *albopictus* larvae or/and pupae against the total number of houses examined.
- **Larvae Density (LD):** Total number of *Ae*. *aegypti* or/and *Ae*. *albopictus* larvae over the total number of containers examined.
- **Pupae Density (PD):** Total number of *Ae*. *aegypti* or/and *Ae*. *albopictus* pupae over the total number of containers examined.
- **Ovitrap Index (OI):** Percent positive ovitraps against the total number of ovitraps recovered for each ovitrap surveillance.

#### Larviciding with *Bacillus thuringiensis israelensis* (Bti)

VectoBac WG, a 3000 ITU/mg Bti formulation manufactured by Valent BioSciences LLC, USA was used in this study. The Bti treatments were conducted from EW 24–35 in Z-7L by the vector control staff from the Sibu Health Office. In the first 4 weeks (EW 24–27) weekly treatments were conducted. This was followed by fortnight treatments for the subsequent 8 weeks (EW 28–35).

The Bti treatments were by direct application and by wide area spray application.

The direct application at 8 g/1000 L targeted all indoor and outdoor in-use artificial containers within the house compound. A calibrated spoon was used to introduce the Bti formulation into the containers. The treated containers were immediately marked with permanent paint.

The wide area spray application at 500 g/ha targeted all outdoor containers. The spray mix was prepared as according to the manufacturer’s recommendation at 125 g in 12 L water. The mist of Bti droplets was dispersed by 3 applicators, each using a backpack motorized mist blower unit, Stihl SR 420. The spraying was conducted between 0830–1130 hours. Before the first spray treatment was initiated, the applicators were trained to do the following: to identify the larval habitats of dengue vectors in the site; the method of holding the spray pipe of the mist blower to provide optimum coverage of the larval habitats; and to use the suitable metering knob to ensure enough Bti mix to treat the larval habitats. The applicators were guided through during the first 2 treatments. The applicators were also provided with a detailed map of the site, with the guidelines on the number of tank mixes to be sprayed for each track in the treatment site.

#### Evaluation of sprayed Bti droplets

A pre-treatment spray application exercise was conducted to determine whether the sprayed Bti droplets were able to penetrate the larval habitats in the dense undergrowth and to reach habitats in the higher levels of the buildings. Twenty dry transparent plastic containers (17cmx10cmx4cm) without lids were placed randomly without the applicator’s knowledge. The containers were placed on the ground amid any other water storage containers, outdoor furniture and dense vegetation. Containers were also placed in the first level of the houses, behind walls skirting the staircases and balcony. The applicator sprayed the Bti mix in the study site. The containers were then collected and covered with lids. The efficacy of the sprayed droplets in the containers was tested at the Institute for Medical Research (IMR), Malaysia. At IMR, 200 mL water and 10 *Ae*. *aegypti* (L3) larvae were introduced into each container. Mortality was recorded at 3–5 hours, 24 hours and 48 hours post larvae exposure. The residual efficacy of the deposited Bti droplets in the containers was also measured on 7, 14, 21 and 28 days post spraying. On all these days a batch of 10 larvae were introduced and mortality was measured as described above.

#### Year 2015, evaluation of the impact of Bti treatment on dengue cases

All suspected dengue cases, detected through active and passive case surveillance from clinics and hospitals, are notified to the nearest district health office. This mandatory action is provided under the Prevention and Control of Infectious Diseases Act 1988. Serologically confirmed dengue cases (NS1/IgM/IgG positive) are registered after investigation. The weekly epidemiology data were extracted from the Sibu district health office for sites Z-5 and Z-14 (the control sites) and site Z-7L (Bti treated site) for the years 2014 and 2015.

Z-5, Z-7L and Z-14 had 1809, 2640 and 2,227 houses, respectively. We determined the population in the 2 study sites by multiplying the number of houses with 4.2, the average number of individuals in a household in Sarawak. This average number was determined by the Institute for Public Health through National Health and Morbidity Survey conducted in 2011 for each of the 15 States in Malaysia [20]. The population numbers were then used to determine the disease Incidence Rate (IR) per 100,000 population.

### Year 2016, impact of wide area Bti spray treatment integrated in the operational dengue control program

In 2016, the Sibu district health office implemented the operational routine dengue control program activities in sites Z7-L, Z-5 and Z-14 from EW 9/2016. The activities were based on the guidelines as outlined by the Vector Borne Disease Control Program [8]. Adulticiding by thermal fogging and truck mounted ULV misting with PY and OP insecticides were conducted within 400 m radius from the case house within 24 hours of reporting. The adulticiding control activity was conducted once every 3 days in these 3 sites.

The outdoor premise of the case house and of the surrounding houses within 100 m from the case house were larvicided with Bti (VectoBac WG). The applicators used backpack mist blowers (Stihl SR 420) to target the larvicide spray mist onto the larval habitat as they came across the habitats. The larviciding exercise was conducted weekly for the first 4 weeks, followed by once in 2 weeks until no new cases were reported within the 400 m radius from the case house. All residents of the case house and neighbouring homes within the 400 m radius from the case house were supplied with temephos 1% SG (Abate 1SG) to treat septic tanks and in-use water storage containers. The control activity also included the search and destruction of all potential larval habitats found indoors and outdoors within 400 m radius from the case house, and covered as much as 80% of the indoors and outdoors. As soon as the dengue case was serologically confirmed, the indoors of the case house was aerosol treated with a water-base insecticide to kill any infected *Aedes* spp mosquitoes.

The dengue incidence rate continued to escalate despite the above-mentioned operational dengue control activities in all the 3 sites. So, the wide area Bti spray application was included in the routine vector control program for site Z-7L only from EW 27/2016. The wide area spray application covered the entire 12 ha of site Z-7L and it was conducted fortnightly. It was conducted just as described in the section above under “Larviciding with *Bacillus thuringiensis israelensis* (Bti)”. This control activity continued until the end of the year.

The disease incidence rate (IR) per 100,000 population was determined for sites Z-7L Z-5 and Z-14 for the year 2016 just as described in the section under “Year 2015, evaluation of the impact of Bti treatment on dengue cases”.

#### Statistical tests

In the pilot operational field efficacy study in 2015, the impact of Bti treatment on *Aedes* spp mosquito population was determined by making comparisons between the treated (Z-7L) and the control (Z-5) sites for *Aedes* index (AI), larval density (LD) and ovitrap index (OI). The comparisons were made between the sites at 3 different phases: pre Bti treatment phase (EW 19–23); Bti treatment phase (EW 24–35); and post Bti treatment phase (EW 36–41). All analyses were conducted using IBM Statistical Package for Social Science software (SPSS) ver. 25. Shapiro-Wilk test was used for normality presumption (p<0.05). When any one set of the data, either from the treated or the control zone was not normally distributed the non-parametric Mann-Whitney U test was performed to determine the differences in the parameters mentioned above between the sites. As for normally distributed data, the t-test was used to determine the differences between the sites (p<0.05).

A correlation between the AI and LD between the sites and within each site at the 3 different study phases was determined using the Spearman correlation coefficient at p<0.05. The regression analysis between AI and LD was performed to clarify the relationship and r^2^ was determined.

The correlation between OI and AI was also determined for the corresponding week in each site. Pearson correlation coefficient at p<0.05 was used to determine the correlation of the parameters.

## Results

### Pilot operational field efficacy study in the year 2015

#### Evaluation of sprayed Bti droplets

In the pre-treatment spray application exercise, a 97–100% larval mortality was observed in all the 20 containers that were placed randomly outdoors in varied locations in site Z-7L. The larval mortality was observed in all the containers within 3–5 hours of exposure to the sprayed Bti droplets, irrespective of the location of the test containers, whether from dense undergrowth or from the upper levels of the premise. This near complete mortality was observed for 21 days post treatment in the larvae population that was introduced weekly into the containers. The larval mortality drastically reduced to 38% in the 28 days post treatment samples. This confirms the residual efficacy of the sprayed Bti droplets for 21 days post treatment in treated waters. Our findings concur with other studies on the residual efficacy of sprayed Bti droplets in the treated habitats for at least 14 days post treatment [21–23].

#### Year 2015, Bti treatment in site Z-7L

A total of 8 Bti treatments were conducted over 12 weeks by direct and spray applications. The direct Bti application into in-use water storage containers was conducted in homes where the owners were present and gave their consent. In the treatment area of 2640 houses, 440 homeowners consented to allow Bti treatment in their in-use containers, i.e. 16.67% of the household. The 440 homes had 1408 containers, and we were allowed to treat 70% (985) of the containers.

The wide area Bti spray application covered the entire outdoor premises of all the 2640 houses, targeting the natural and artificial containers. The 12 ha site required 5.25 kg of Bti in 504 L water per spray treatment. The spray mist covered all potential and actual outdoor larval habitats, which included natural containers comprising leaf axils, curled dry leaves, tree holes, vegetation and artificial containers such as animal feeds, concrete and earth drains, sunken ground below the houses, discarded containers, garbage dumping site and roof gutters.

### Year 2015, impact of Bti treatment on *Aedes* index, larvae-pupae indices and ovitrap index

#### Aedes index and larvae-pupae indices

The impact of the direct Bti application into the water storage containers was measured using the *Aedes* index (AI) and *Aedes* spp larvae-pupae density. The impact of the outdoor treatment into cryptic containers via wide area Bti spray application was measured using the ovitrap index. The outdoor environmental condition of the site was not favourable for the entomology team to attempt larvae and pupae surveillance of the cryptic containers.

The weekly surveillance was conducted over 23 weeks (EW 19–41). A total of 1,831 and 1,969 containers were surveyed in sites Z-7L and in Z-5, respectively. The mean number of in-use containers surveyed per household was similar in both study sites during each phase of study (p>0.05) (Table 1).

**Table 1:** Year 2015, the mean number ± S.E. of in-use containers surveyed per household in epidemiology weeks (EW) 19–41 in Z-5, control site, and in Z-7L, Bti treated site.

Study Phase	Epi Weeks (number of surveillance)	Mean Number of Containers ± S.E. per Household	
		Z-5 Control site	Z-7L Bti treated site
**Pre-Treatment**	19–23 (5)	7.48 ± 0.69 ^a^	7.34 ± 0.57 ^a^
**Weekly Bti Treatment**	24–27 (4)	6.13 ± 0.41 ^b^	5.90 ± 0.27 ^b^
**Fortnightly Bti Treatment**	28–35 (8)	5.64 ± 0.47 ^c^	5.15 ± 0.27 ^c^
**Post Treatment**	36–41 (6)	4.77 ± 0.36 ^d^	5.28 ± 0.52 ^d^

^a,b,c,d^ Same letters within each study phase indicated no significant difference (p>0.05) of the mean number of containers surveyed between the two sites Z-5, the control site, and Z-7L, the Bti treated site.

It was noted that site Z-7L had pre dominantly *Ae*. *aegypti* population, 5.3 folds more than *Ae*. *albopictus*, while site Z-5 was pre dominantly *Ae*. *albopictus*, 2.4 folds more than *Ae*. *aegypti*.

**Fig 2:**
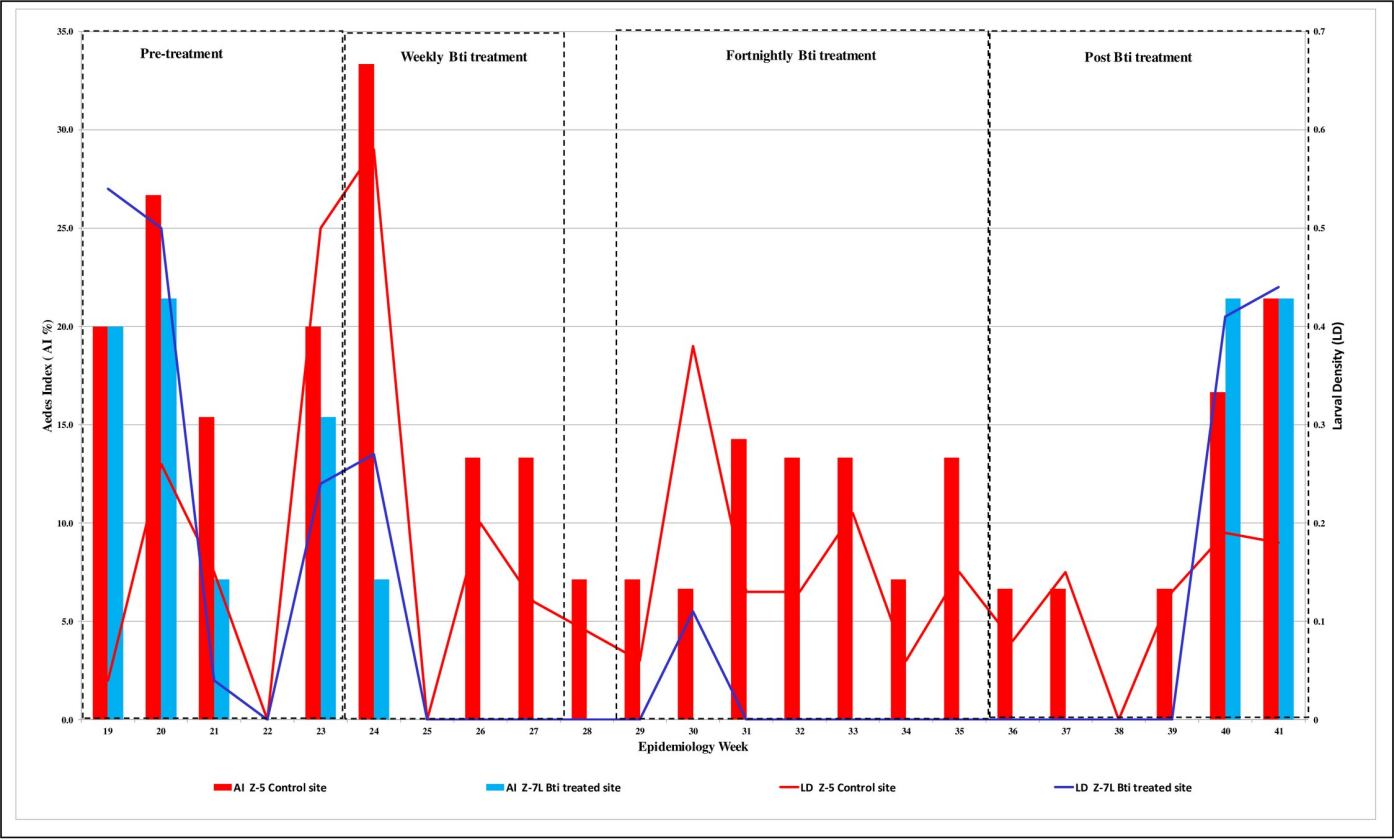
For 2015 epidemiology weeks 19–41, *Aedes* index (AI, %) and Larval Density (LD) from Z-5, the control site, and from Z-7L, the Bti treated site.

**Fig 3:**
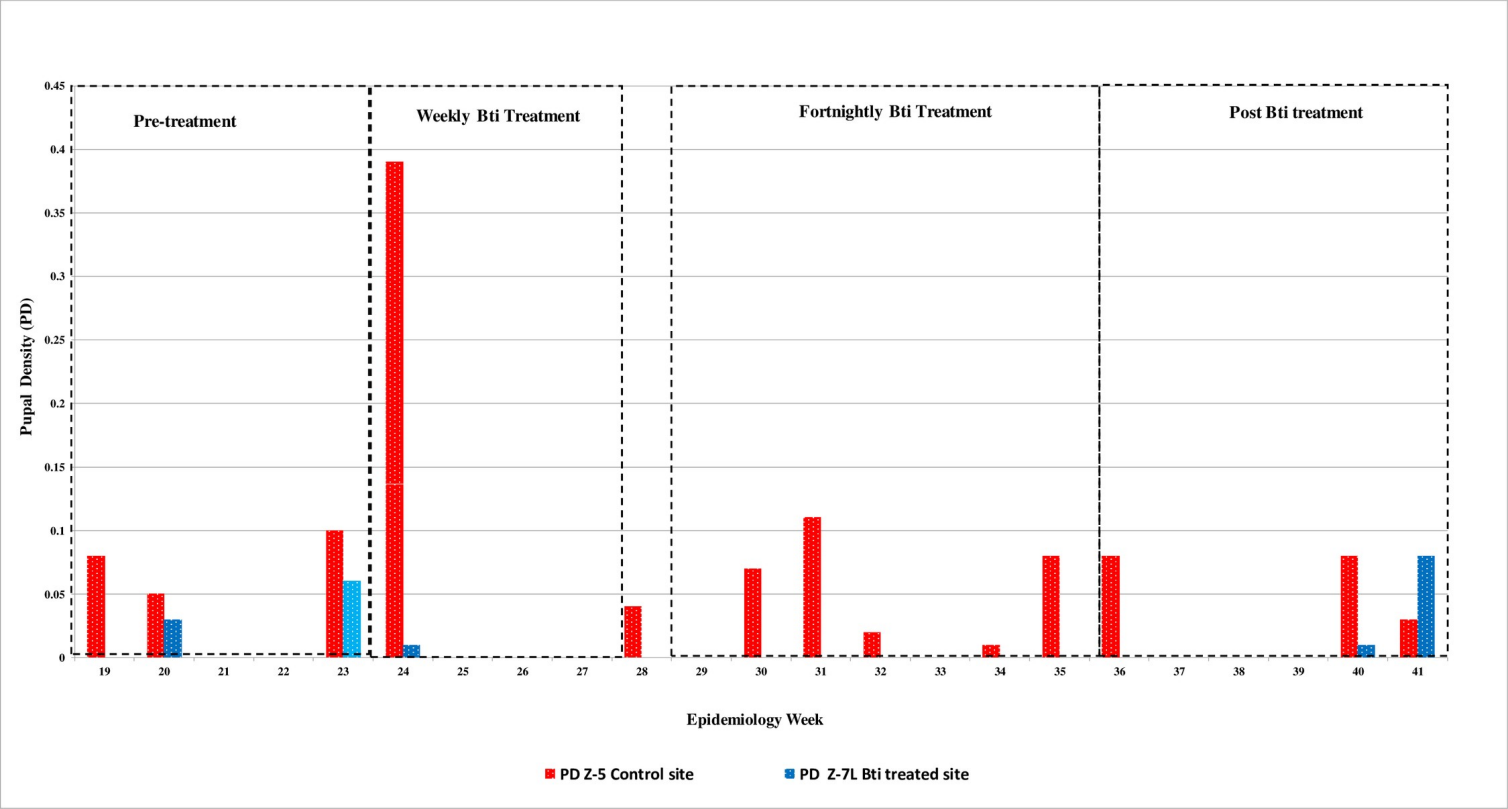
For 2015 epidemiology weeks 19–41, pupa density (PD) from Z-5, the control site, and from Z-7L, the Bti treated site.

*Aedes* index (AI)and larvae density (LD) are as shown in Fig 2 and pupa density (PD) in Fig 3.

Throughout the trial duration of 23 weeks, the control site, Z-5, had AI above the national threshold values to implement dengue vector control, i.e. >1% for 20 weeks and pupae were observed for 13 weeks. In the Bti treated site, Z-7L, the AI was above the threshold value during the pre-treatment phase and 5 weeks after the Bti treatment ended. There were no larvae and no pupae production in the Bti treated in-use containers during the treatment phase and in the first 4 weeks of the post treatment phase. The containers were re colonized 5 weeks after the Bti treatment ended.

The mean AI (± S.E.) in both study sites are as shown in Table 2. There was no significant difference between the 2 sites during the pre-treatment phase (p>0.05). The AI in site Z-7L significantly lowered as soon as the Bti treatment was initiated, and no larvae were then observed in the in-use containers throughout the Bti treatment phase for a continuous 11 weeks and for another 4 weeks in the post treatment phase (p<0.05).

**Table 2:** For 2015, the mean *Aedes* index (AI) ± SE per surveillance conducted in epidemiology weeks (EW) 19–39 in Z-5, control site and in Z-7L, Bti treated site. Impact of larviciding with Bti on *Aedes* index was determined at p = 0.05.

Study Phase	Epi Weeks	Z-5	Z-7L
	(number of surveillance)	Control site	Bti treated site
**Pre-Treatment**	19–23	16.42 ± 4.50 ^a^	12.78 ± 4.06 ^a^
	(5)		
**Bti Treatment**	24–35	11.84 ± 2.32 ^a^	0.59 ± 0.59 ^b^
	(12)		
**Post Bti Treatment**	36–39	5.03 ± 1.70 ^a^	0 ^b^
	(4)		

^a,b^ Same letters within each study phase indicated no significant difference (p>0.05) of the mean *Aedes* Index (AI) between the two sites Z-5, the control site, and Z-7L, the Bti treated site, and different letters within each study phase indicate that means were significantly different (p<0.05).

The mean LD (± S.E.) in both study sites are as shown in Table 3. The *Aedes* spp population was significantly lower in the Bti treated site, Z-7L, during the treatment phase and for 4 weeks after post treatment in comparison to the pre-treatment phase (p<0.05). Between the 2 study sites, the LD was significantly lower in the Bti treatment site during the Bti treatment phase and for the next 4 weeks in the post treatment phase (p<0.05).

**Table 3:** For 2015, the mean larval density (LD) ± SE per surveillance conducted in epidemiology weeks (EW) 19–39 in Z-5, control site and in Z-7L, Bti treated site. Impact of larviciding with Bti on LD was determined at p = 0.05.

Study Phase	Epi Weeks	Z-5	Z-7L
	(number of surveillance)	Control site	Bti treated site
**Pre-Treatment**	19–23	0.19 ± 0.09 ^a^	0.26 ± 0.11 ^a^
	(5)		
**Bti Treatment**	24–35	0.16 ± 0.05 ^a^	0.03 ± 0.02 ^b^
	(12)		
**Post Bti Treatment**	36–39	0.09 ± 0.03 ^a^	0 ^b^
	(4)		

^a,b^ Same letters within each study phase indicated no significant difference (p>0.05) of the mean Larval Density (LD) between the two sites Z-5, the control site, and Z-7L, the Bti treated site, and different letters within each study phase indicate that means were significantly different (p<0.05).

In each study site the AI was correlated to LD. The AI and LD had a strong positive significant correlation in Z-7L, the Bti treated site (r = 0.93; p<0.05) and a moderate positive significant correlation in Z-5, the control site (r = 0.62; p<0.05).

**Fig 4:**
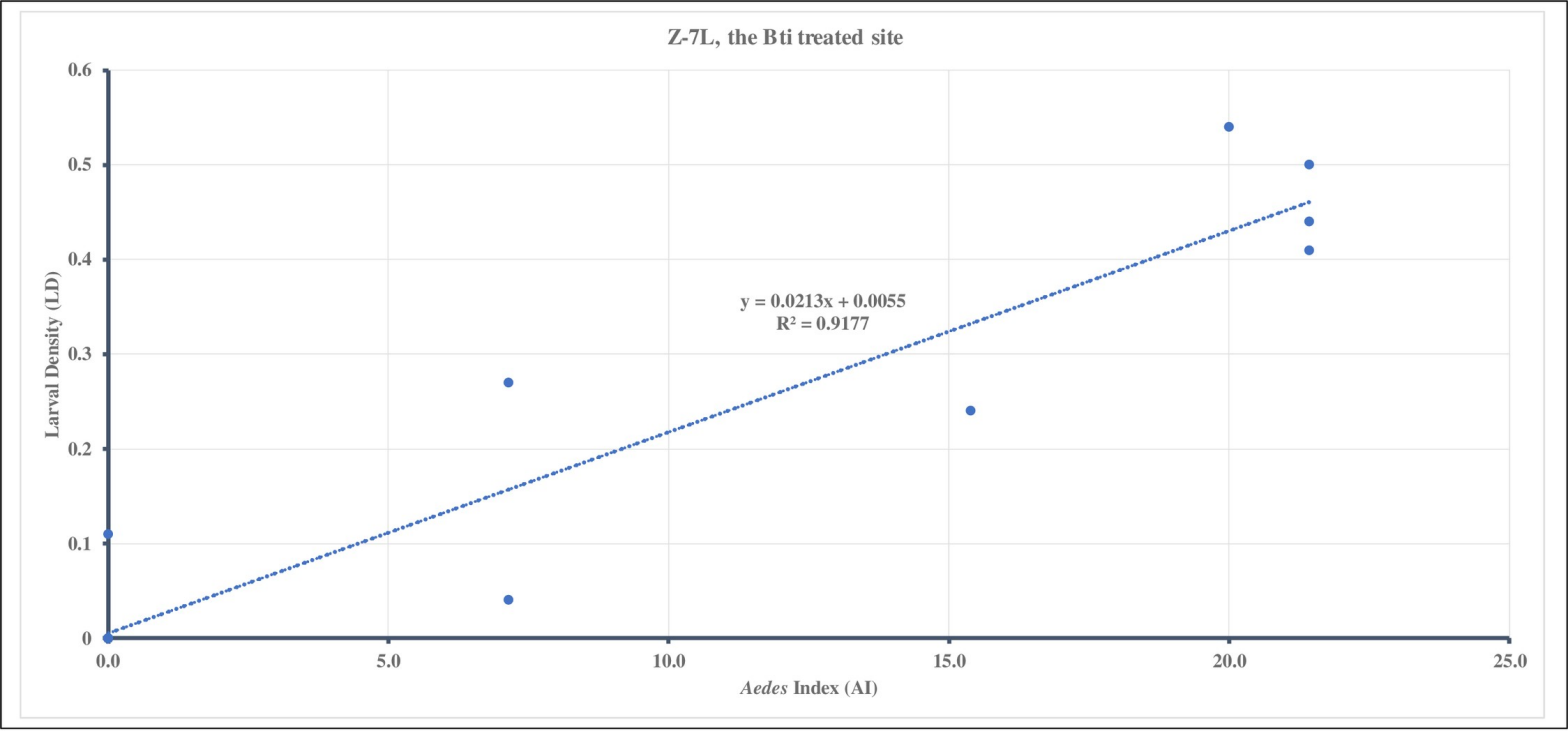
For 2015, the regression relationship between larval density (LD) and *Aedes* index (AI) during the 23 weeks of entomological surveillance conducted in Z-7L, the Bti treated site. There was a strong positive correlation (R^2^ = 0.92) between LD and AI.

**Fig 5:**
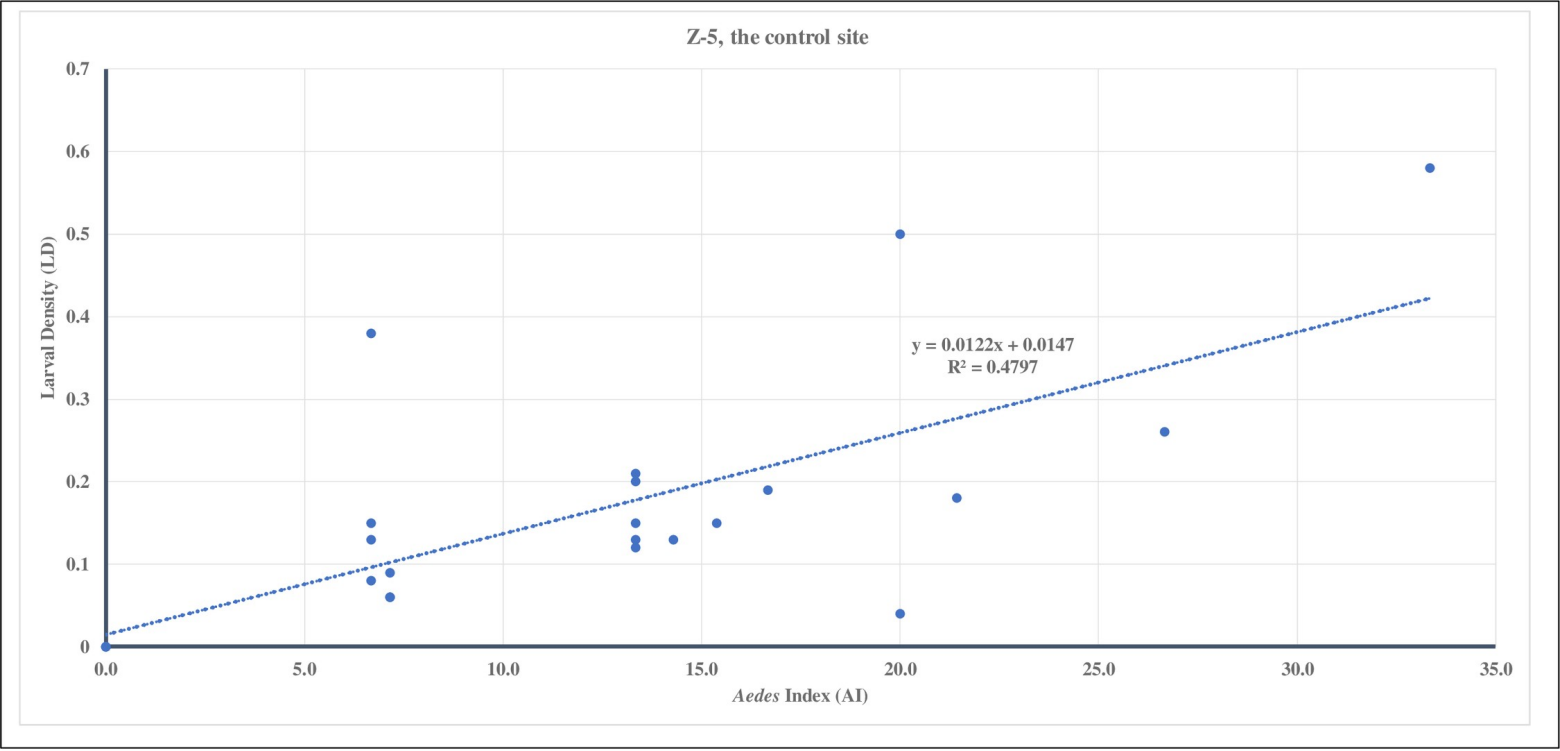
For 2015, the regression relationship between larval density (LD) and *Aedes* index (AI) during the 23 weeks of entomological surveillance conducted in Z-5, the control site. There was a moderate positive correlation (R^2^ = 0.48) between LD and AI.

The regression relationship (R^2^) between AI and LD in Z-7L, the Bti treated site, was 0.92 (Fig 4) and it was higher than in Z-5, the control site at 0.48 (Fig 5).

The strong positive correlation between AI and LD in the Bti treated site, Z-7L, was throughout the 23 weeks of study (Table 4). As for the control site, Z-5, the strong positive correlation was only observed towards the later part of the study from EW 36–41 (Table 4).

**Table 4:** Correlation coefficient between *Aedes* index (AI) and larval density (LD) within Z-5, control site and in Z-7L, Bti treated site.

Study Phase	Epi Weeks		Z-5 Control Site	Z-7L Bti Treated Site
**Pre-treatment**	19–23	Correlation Coefficient	0.667	0.900
		P-value	0.219	0.037*
**Treatment**	24–35	Correlation Coefficient	0.554	0.739
		P-value	0.062	0.006*
**Post-treatment**	36–41	Correlation Coefficient	0.880	0.980
		P-value	0.021*	0.001*

* Indicates *Aedes* index (AI) and larval density (LD) are significantly correlated (p<0.05).

#### Ovitrap index

For the entire study period, 11 sets of ovitrap surveillance were conducted in each study site. The pre-treatment ovitrap surveillance showed the presence of both *Ae*. *aegypti* and *Ae*. *albopictus* populations in the Bti treated site (Z-7L), while the control site (Z-5) had only *Ae*. *albopictus*.

The mean OI (± SE) in both study sites are as shown in Table 5.

**Table 5:** For 2015, the mean ovitrap index ± SE per surveillance conducted in epidemiology weeks (EW) 19–41 in Z-5, control site, and in Z-7L, Bti treated site. Impact of larviciding with Bti on OI was determined at p = 0.05.

Study Phase	Epi Weeks	Z-5	Z-7L
	(number of surveillance)	Control site	Bti treated site
**Pre-Treatment**	19–23	6.70 ± 3.30 ^a^	9.03 ± 3.90 ^a^
	(3)		
**Bti Treatment**	24–35	21.10 ± 4.30 ^a^	8.64 ± 2.30 ^b^
	(5)		
**Post Bti Treatment**	36–41	45.60 ± 5.90 ^a^	19.70 ± 5.00 ^b^
	(3)		

^a,b^ Same letters within each study phase indicated no significant difference (p>0.05) of the mean Ovitrap Index (OI) between the two sites Z-5, the control site, and Z-7L, the Bti treated site, and different letters within each study phase indicate that means were significantly different (p<0.05).

In control site Z-5, the OI rose steeply with time during the study period. An initial OI of 6.7 ± 3.3 increased to 21.1 ± 4.3 during the EW 24–35 and further escalated to 45.6 ± 5.9 in the following weeks, EW 36–41. However, in the treated site, Z-7L, the OI was suppressed and it was significantly lower in comparison to the control site, Z-5 (p<0.05). Moreover, during the Bti treatment phase, the OI in the treated site was less than the national threshold value of 10% to initiate any dengue vector control measure [14]. The OI was observed to increase during the post treatment phase. However, the OI in the treated site was significantly lower than the control site in the post treatment phase (p<0.05).

In each study site the 11 sets of OI had moderate, but not significant correlation to AI (p>0.05) with r = 0.39 in Z-7L, the Bti treated site and r = 0.30 in Z-5, the control site.

#### Year 2015, impact of Bti treatment on dengue cases

**Fig 6:**
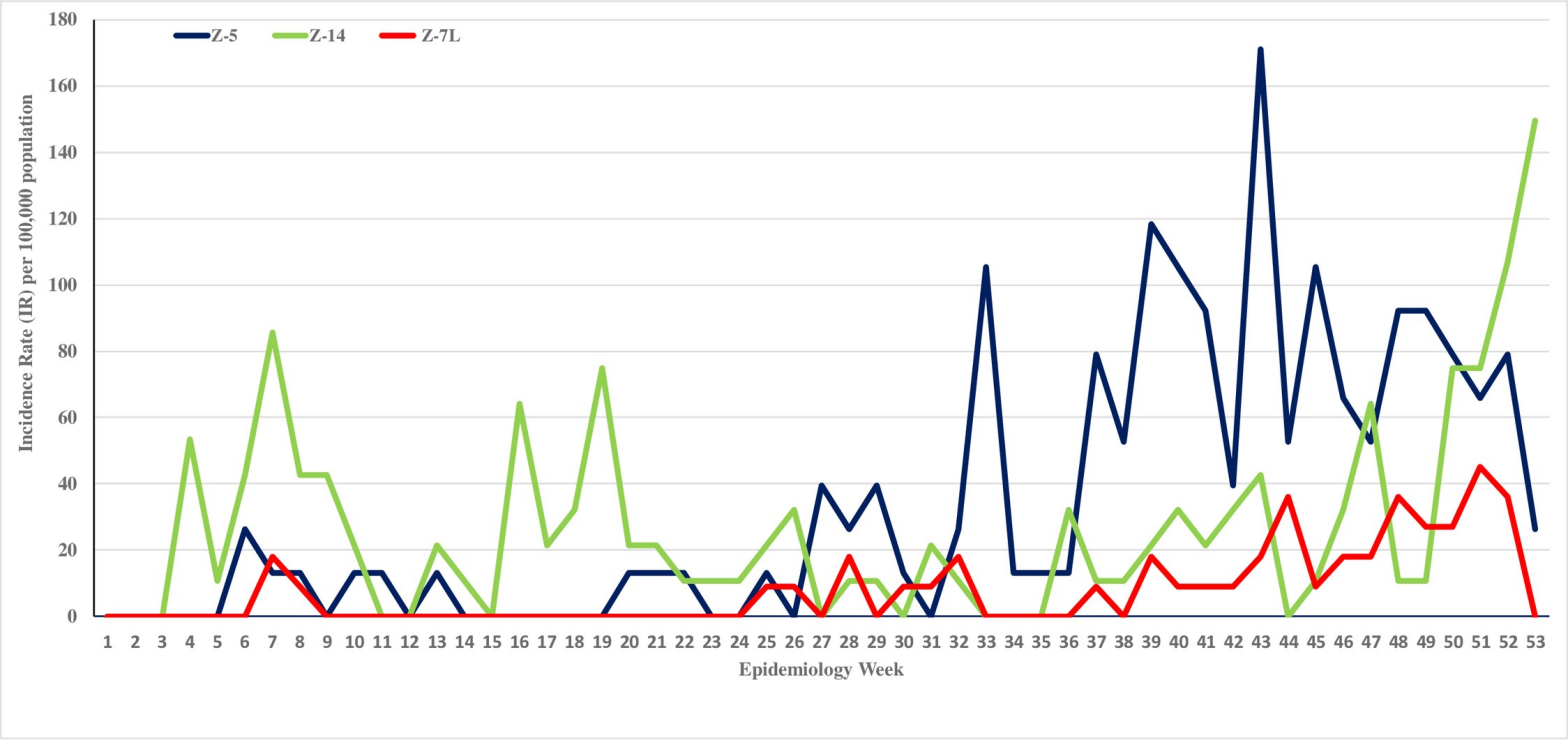
For 2014 epidemiology weeks 1–53, the incidence rate (IR) per 100,000 population in 3 sites: Z-5, Z-7L and Z-14. In all 3 sites routine operational vector control activities were conducted, excluding larviciding with Bti.

**Fig 7:**
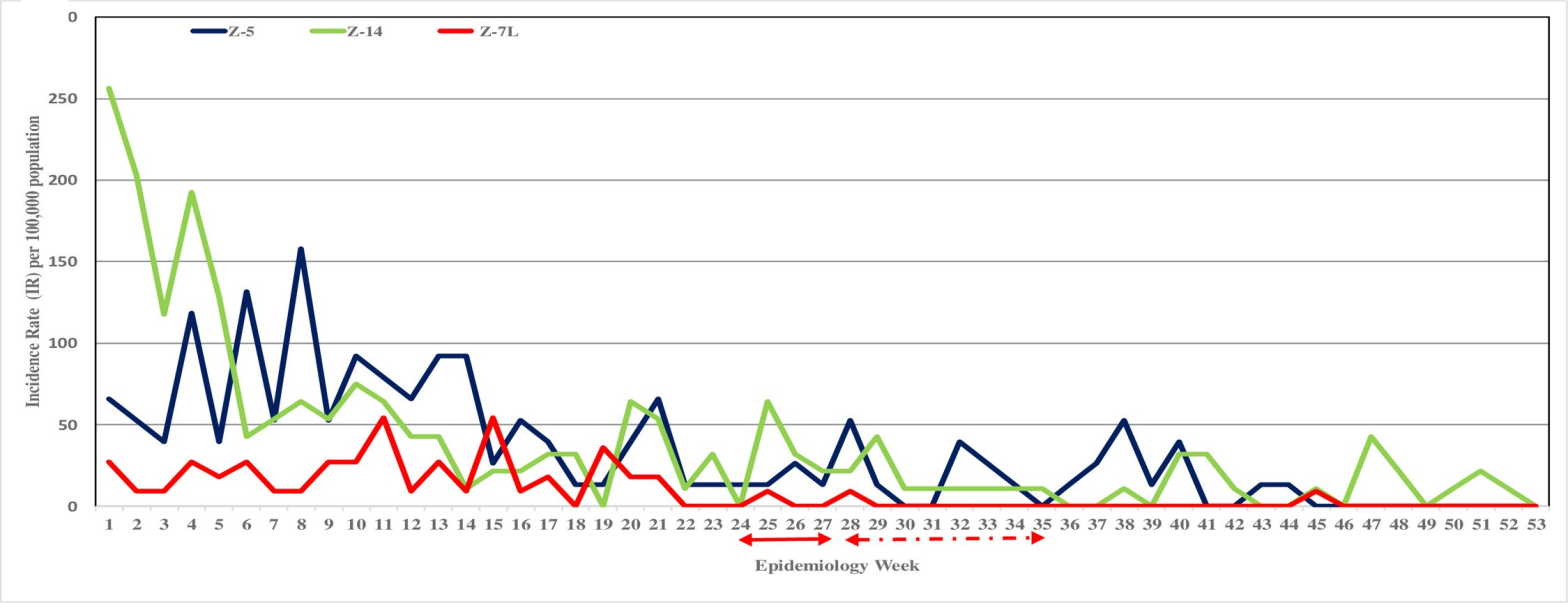
For 2015 epidemiology weeks 1–52, the incidence rate (IR) per 100,000 population in 3 sites: Z-5, Z-7L and Z-14. In all 3 sites routine operational vector control activities were conducted. However, the direct Bti application into containers and wide area Bti spray treatment were integrated in the routine control activities in site Z-7L on weekly interval from EW 24–27 (↔) and fornightly interval from EW 28–35 (←⋅→).

Dengue cases in sites Z-5, Z7-L and Z-14 for the years 2014 and 2015 are presented as incidence rate (IR) per 100,000 population for epidemiology weeks (EW) 1–53 (Figs 6 and 7). The combined incidence rate for the different periods in 2015 is as presented in Table 6.

**Table 6:** For 2015–2016, the combined incidence rate (IR) per 100,000 population during the pre Bti treatment impact and Bti treatment impact phases. In 2015, the Bti treatment was implemented in Z-7L in EW 24 and in 2016 in EW 27. Impact on the dengue transmission was observed 4 weeks after the Bti treatment initiation.

Year	Epidemiology Week EW	Incidence Rate (IR) per 100,000 population		
		Z-7L	Z-5	Z-14
**2015**	**1–28**	460	1527	1753
	**29–52**	9	263	310
**2016**	**1–31**	577	855	1304
	**32–52**	99	290	492

In 2014, all 3 sites had dengue cases occurring from the first quarter of the year. An increase in the dengue incidence was observed from EW 25 until the end of the year (Fig 6). The 29 weeks (EW 25–53) of episode had a total IR ranging between 397–1671/100,000 population among the 3 sites. Disease transmission continued into 2015 and it was persistent in all the 3 sites (Fig 7). The Bti treatment was integrated into the routine vector control activities from EW 24–35 in Z-7L. The impact of the larviciding with Bti on the dengue cases was measured 4 weeks after the initiation of the Bti treatment. The 4 weeks, EW 25–28, was classified as the window period, where the surviving female adult mosquitoes were still able to transmit the dengue virus [24]. There was no reported dengue case in Z-7L from EW 29, during the fortnightly Bti treatment phase until EW 35 and beyond into post Bti treatment phase. A single case was reported in EW 45, and after this no more case was reported until the end of the year. Thus, a 51 fold reduction in the IR/100,000 population was observed during EW 29–52 compared to EW 1–28 (Table 6). During the same period of time (EW 24–35) in the control sites, Z-5 and Z-14, routine vector control activities excluding Bti treatments were conducted. A 6 fold reduction in the IR was observed from EW 29–52 in the 2 control sites (Table 6), and almost every week for the year there were dengue cases in Z-14 and until EW 44 in Z-5 (Fig 7).

### Year 2016, impact of wide area Bti spray treatment integrated in the operational dengue control program

**Fig 8:**
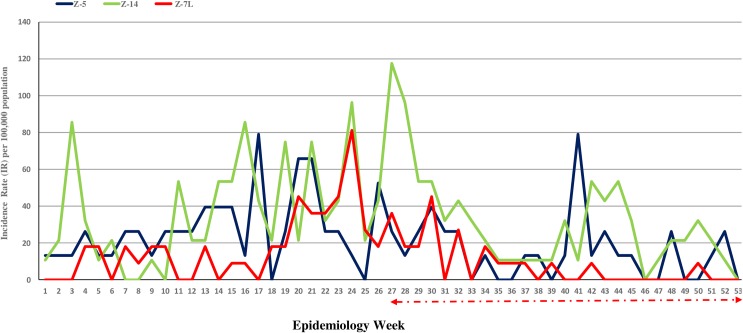
For 2016 epidemiology weeks (EW) 1–52, incidence rate (IR) per 100,000 population in 3 sites: Z-5, Z-7L and Z-14. In all 3 sites routine operational vector control activities were conducted, excluding wide area Bti spray treatment. However, in site Z-7L as from EW 27 until the end of the year (←⋅–⋅–⋅–⋅→) the wide area Bti spray application on fortnight intervals was integrated into the routine operational vector control activities.

In 2016, the dengue cases resurfaced in EW 4 in site Z7-L, twenty-one weeks after Bti treatment was terminated in EW 35/2015. The dengue transmission was persistent in all the 3 sites, Z-7L, Z-5 and Z-14 (Fig 8). The Sibu Health Office activated vector control activities from EW 9, but, the IR continued to escalate in all the sites. To combat the increasing dengue incidence from EW 27–52, the wide area Bti spray application was reintroduced in site Z-7L. The wide area spray application covered the entire 12 ha site of Z-7L and was conducted on fortnight intervals. The case numbers then declined evidently from EW 35. No successive cases were recorded after EW 37 until the end of the year, except for a single case in EW 39, EW 42 and EW 50. A 6 fold reduction in the IR/100,000 population was observed during the Bti treatment impact phase (EW 32–52) compared to EW 1–31 (Table 6). While the other 2 non Bti sites, Z-5 and Z-14, had no reprieve from dengue cases throughout the year from EW 1, particularly the community in Z-14. A 3 fold reduction in the IR/100,000 population was observed from EW 32–52 in the 2 control sites (Table 6).

## Discussion

In the state of Sarawak, from 2014 to 2016, Sibu District contributed to a minimum of 50% of the cases in Sarawak State and within the Sibu District an average of 26% of the cases was from site Z-7L [17]. The Sibu Divisional Health Office (DHO) carried out intensive adulticiding from EW 25/2014. They also conducted larviciding with temephos SG, temephos 500E and Bti aqueous suspension formulation. Despite the intense vector control activities, there was no let-up in the active transmission of the dengue virus for a stretch of 57 weeks (EW 25/2014 –EW 28/2015). Several factors could have hampered the realisation of the vector control activities. First, it could be due to partial susceptibility of the wild *Aedes* vector population to temephos and adulticides. Routine monitoring has shown that *Ae*. *aegypti* and *Ae*. *albopictus* from Sibu had only 87% and 52% larval mortality at WHO diagnostic dosage to temephos, respectively [13]. In 2015, it was documented that wild *Ae*. *albopictus* population from Sibu exhibited different resistance levels to varying insecticides from the 4 major classes of insecticides [14]. Secondly, it could be due to the presence of high numbers of containers in the outdoors. Most of the containers are discards amid thick ground vegetation. The drainage system consisting of earth and concrete drains in this site was also generally blocked by discard containers. All outdoor containers exposed to rain or sewer water provide a suitable harbourage for the colonization of dengue vectors [25–28]. In the larvae survey conducted in 2015–2016 in 4 ongoing dengue hotspot sites in Malaysia, it was identified that the highest number of positive containers were found outdoors and these outdoor containers were 3.5 folds more productive than indoor containers. Most of the outdoor containers were plastics and the locality with the highest ratio of container numbers per hectare had persistent numbers of dengue cases over the years [5].

In 2015, in the midst of mayhem caused by active dengue transmission in 3 sites, Z-7L, Z-5 and Z-14, the Sibu DHO decided to evaluate larviciding with Bti via direct application into stored water containers and wide area spray application into all outdoor containers in site Z-7L. The entomology team comprised 4 personnel, conducted the weekly larvae-pupae surveillance, ovitrap surveillance and the direct application of Bti treatment into potable containers. They also carried out the lab works on the colonization and identification of all collected specimens. Due to minimal workforce covering a wide range of activities for 23 continuous weeks, we had to limit the larvae-pupae surveillance to 15 houses per site, total 30 houses from the 2 study sites, Z-7L and Z-5. Even though, it was 15 houses per site, but the number of containers examined in these 15 houses every week was large, it was in the range of 75–120 containers. So, a total of 1,831 and 1,969 containers were examined in Z-7L and Z-5, respectively during the 23 weeks of entomological surveillance. Also due to limited personnel, for the ovitrap surveillance we restricted the number of ovitraps per site to the minimum number as outlined in the national guidelines, i.e. 30 ovitraps per site [8].

Prior to the introduction of wide area spray application of Bti from backpack misters, the outdoor containers in site Z7-L were larvicided with temephos 500E or Bti aqueous suspension formulation using a knapsack compression sprayer. The knapsack compression sprayer enabled the applicator to do only spot-treatment, whereby as they came across the containers they sprayed onto the containers. The knapsack compression sprayer with the fan nozzle has a limited dispersal swath of 1.5 m from the applicator. Thus, in site Z7-L this method of treatment was not able to disperse the larvicide droplets into the larval habitats found in this site: large number of containers that were sheltered from sunlight beneath dense vegetation; discarded containers that were piled as heaps in the scattered garbage dumping sites; stagnated water in the crevices beneath the cement floor of the houses; containers found below the drain covers; and roof-gutters. Hence, in the 2015 pilot study in Sibu the wide area larvicide spray treatment was used to disperse the Bti droplets from a backpack motorized sprayer to provide a complete coverage of the larval habitats found outdoors in all the 2640 houses in site Z-7L. The pre-treatment exercise in this study confirmed that the Bti droplets were able to penetrate through barriers and deposit into water receptacles in dense vegetation at ground level and at first level of the building, which was about 5 meter from the ground. The Bti droplets were efficacious for 21 days post treatment with 97–100% larval mortality. This same sprayer model used in Sibu was used in northern Queensland, Australia and it was reported to disperse Bti micro-droplets of average volume median diameter (VMD) of 63 um into 16 m of dense bushland without efficacy decline over distance [26].

The effectiveness of the wide area Bti larviciding from backpack motorized sprayer in site Z-7L was furthermore proven by the suppression of the ovitrap index during the Bti treatment phase in the year 2015. The ovitrap index (OI), which is used to measure the relative abundance of wild *Aedes* adult mosquitoes [19], indicated the significant suppression of the adult mosquitoes in the Bti treated site by 2.4 folds compared to the control site, Z-5. The OI in site Z-7L during the Bti treatment phase was suppressed to less than 10%, the threshold value to initiate any vector control activity as per the national guidelines [8]. Similar OI result was observed, suppressed to below 10%, when wide area Bti spray application was conducted in a dengue endemic residential site in Shah Alam, Selangor State, Malaysia [29]. The OI at the control site in Shah Alam remained at more than 40% during the evaluation period.

In the 2015 pilot study, the direct and wide area spray applications of Bti were both implemented in site Z-7L. The direct Bti application was used to treat the water storage containers found principally in the indoors of the houses. This treatment method covered a very low percentage (16.7%) of the household, as the owners were not in or were reluctant to the larvicide treatment. Nevertheless, the strong positive correlation between AI and LD in the Bti treated site, Z-7L, (p<0.05) indicates that the surveillance and the Bti treatment program covered all the containers found in the household which was receptive to the Bti treatment. The treated containers were devoid of *Aedes* spp immatures throughout the treatment phase and for 4 weeks into the post treatment phase. The wide area Bti spray application covered almost the entire outdoor larval habitats in the 12 ha site of site Z-7L. There was significant suppression in the OI (p<0.05). However, the OI and AI had only a moderate, but insignificant correlation (p>0.05). Thus, indicating that the productive containers for *Aedes* spp adults are not just the in-use potable containers, but also the widespread containers found in the outdoors. The treated in-use potable containers were not productive throughout the treatment and post treatment phases. So, the positive OI was most probably contributed by the *Aedes* spp that were colonized in the outdoor containers. In Malaysia, outdoor containers were found to be 3.5 folds more productive than indoor containers [5].

The supplementary Bti treatment in Z-7L, together with routine vector control activities impacted the *Aedes* spp mosquito density and the transmission of the dengue virus. In 2015, four weeks after initiating the Bti treatment, a 51 fold reduction in the IR per 100,000 population was observed, with only one single case in 25 weeks (EW 29–52). So, in 2016 in site Z-7L when dengue cases resurfaced and the numbers continued to escalate despite the intensive adulticiding once every 3 days from EW 9, the wide area Bti spray application was integrated into the routine vector control program from EW 27/2016. Based on the 2015 pilot study data, in 2016 we focused on the treatment of the outdoor larval habitats using the wide area Bti spray strategy and did not conduct the direct application of Bti into water storage containers. Then the dengue case numbers declined evidently with 6 folds reduction in the IR per 100,000 population. No cases were reported within the 11 weeks between EW 39–52. During the same years, 2015–2016, in the control sites, Z-5 and Z-14, there were cases in almost all the weeks from EW 1–52. A 6 and 3 fold reduction in the IR was observed in both sites in 2015 and 2016, respectively. The reduction in the control sites could have been impacted by the intensive adulticiding activities conducted once every 3 days.

In 2015, in site Z-7L the Bti treatment was implemented from EW 24 on weekly intervals for the first 4 weeks, followed by fortnightly treatments for the next 8 weeks. Active transmission was not observed from EW 29. In 2016, in site Z-7L, the interruption to dengue transmission was observed 7 weeks after the initiation of the Bti treatment in EW 27 on fortnight intervals, in addition to the application of routine vector control activities. Multiple cases per week (EW 28–34) were reduced to successive single cases for the next 3 weeks (EW 35–37). After which, a reprieve from active transmission was observed. A longer time span was taken for the impact of the Bti treatment to be evident in 2016 than in 2015, and this could be due to not having a weekly wide area spray treatment at the start of the Bti larviciding program in 2016. The intense weekly treatments for consecutive 4 weeks kill the existing larvae, in addition the larvae that will hatch from eggs oviposited by adults during its 2–4 weeks lifespan and the larvae that will hatch from surviving eggs that have been drying out over a period of several months [24].

The impact of wide area Bti spray treatments have also been noted in USA. In 2016, during the height of local *Ae*. *aegypti* borne transmission of Zika in Miami-Dade County, South Florida, different chemical insecticides sprayed by aerial, truck mounted and backpack had no statistically significant effect on *Ae*. *aegypti* adult counts [30]. The counts were persistently high at >20 per trap per day in the adulticided-only treated area. The mosquito control strategy then shifted to include Bti larviciding in area with ongoing local transmission. The trap counts then declined significantly, and the counts were maintained at 5–10 per trap per day for at least 1 month in the treated site and the zika virus transmission was broken [31]. In 2017, this same County had 29 travel-related Zika cases with no local active transmission. As the rainy season returned, the County performed wide area Bti spray application from truck mounted sprayer as a preventive measure. These larvicide treatments were conducted once in 2 weeks in selected areas [32]. In Key West, Florida the mosquito abatement district conducts weekly aerial Bti applications during the peak wet summer months (May-June), followed by fortnight Bti treatments in July and August to suppress the *Ae*. *aegypti* populations and have successfully prevented local dengue transmissions [28].

Dengue is endemic in Malaysia and it is not confined in urban areas alone. The seroprevalence rates are similar between urban and rural communities [33]. Dengue has major effects on patients’ health and imposes a considerable economic burden in Malaysia. All confirmed dengue patients in Malaysia experienced a drastic decrease in their quality of life during the disease phase [34]. Dengue confirmed patients also experienced chronic fatigue in the post dengue infection phase and the fatigue lasted from months to years [35]. In 2009, the estimated economic burden of dengue illness in Malaysia is US$56 million per year, excluding the cost of prevention, surveillance and dengue vector control activities [35]. In 2010, Malaysia spent US $73.5 million on the National Dengue Vector Control program and 92.2% of this spending was primarily for fogging [36]. Overall, dengue imposes a high economic burden.

In the absence of an effective vaccine, proper vector control is the backbone to prevent dengue outbreaks. Effectiveness of fogging activities could be hampered due to insecticide resistance observed in several states in Malaysia. Wild *Ae*. *aegypti* and *Ae*. *albopictus* mosquitoes are exhibiting varied levels of resistance to all groups of in-use insecticides [37]. Source reduction, covering 400 m from the case house, is another one of the key control measures included in the MOH dengue control program [8], but it is a near impossible feat to search and destroy all the cryptic receptacles. In New Jersey, USA it was found that removing and discarding disposable containers, together with hand-treating container habitats that could not be removed, were less efficient and less cost-effective than motorized backpack applications of Bti [27].

Bti is highly specific affecting only the larvae of mosquito, blackfly and fungus gnats. It has no human toxicity. There is a lack of Bti resistance in field mosquito populations treated for decades with Bti [38]. As demonstrated, the wide area Bti spray applications integrated with or without adulticiding application have shown suppression of adult *Aedes* spp populations and impacted the transmission of diseases [28, 29, 31]. In Malaysia, the Bti application strategy integrated in the national dengue control program can be used prophylactically before the dengue virus outbreak. The frequency of Bti treatment and the timing of the Bti treatment are dependent on the type of larval habitat and the weather condition [39–41]. In areas where it is sunny and rainy throughout the year and the larval habitats are cryptic natural and artificial receptacles, it is recommended that preventive Bti spray treatment to be conducted once every 2 weeks. This strategic measure is to be implemented in selected areas which are densely populated and the *Aedes* indices are above the threshold values, i.e. AI > 1% or OI > 10%. [8]. In areas where the *Aedes* indices are above the threshold values and there is an ongoing dengue transmission, the wide area larvicide spray application is to be conducted every week for 4 weeks, followed by once in 2 weeks treatment.

## Supporting information

S1 Data (PDF)

S2 Data (PDF)

S3 Data (PDF)

## References

[1] Mia MS, Begum RA, Er AC, Abidin RZ, Pereira JJ. Trends of dengue infections in Malaysia, 2000–2010. Asian Pacific Journal of Tropical Medicine. 2013; 462–466. 10.1016/S1995-7645(13)60075-9 23711707

[2] Rose NM. Dengue incidence and the prevention and control program in Malaysia. The International Medical Journal Malaysia. 2015;14(1): 5–9. http://iiumedic.net/imjm/v1/volume-14-no-1-june-2015/.

[3] Lee HL, Hishamudin M. Nationwide *Aedes* larval survey in urban towns of Peninsular Malaysia (1988–1989). Tropical Biomedicine 1990;7: 185–188.

[4] Lee HL. A nationwide resurvey of the factors affecting the breeding of *Aedes aegypti* (L.) and *Aedes albopictus* (Skuse) (Diptera: Culicidae.) in urban towns of Peninsular Malaysia 1988–1989. Tropical Biomedicine 1991;8: 157–160.

[5] Oreenaiza N, Teoh GN, Nazni WA, Seleena B, Lee HL. Identification of *Aedes aegypti* (L) and *Aedes albopictus* (Skuse) breeding habitats in dengue endemic sites in Kuala Lumpur Federal Territory and Selangor State, Malaysia. Southeast Asian Journal of Tropical Medicine and Health. 2017; 48(4): 786–798.

[6] Rozilawati H, Tanaselvi K, Nazni WA, Mohd Masri S, Zairi J, Adanan CR, et al Surveillance of *Aedes albopictus* (Skuse) breeding preference in selected dengue outbreak localities, Peninsular Malaysia. Tropical Biomedicine. 2015; 32(1): 49–64 25801254

[7] World Health Organization (WHO). Global strategy for dengue prevention and control 2012–2020. 2012; 1–35.

[8] Ministry of Health (MOH). Guidelines for dengue control by Vector Borne Diseases Control Program, Ministry of Health, Malaysia. Putrajaya: MOH, 2014.

[9] Cheong WH. The present status of dengue fever/dengue haemorrhagic fever and its control in West Malaysia. Asian Journal of Infectious Diseases. 1978; 2: 136–138.

[10] Ishak IH, Jaal Z, Ranson H, Wondji CS. Contrasting patterns of insecticide resistance and knockdown resistance (kdr) in the dengue vectors *Aedes aegypti* and *edes albopictus* from Malaysia. Parasites & Vectors. 2015; 8: 181–193. 10.1186/s1307-0150797-2 PMCID: PMC4377062. 25888775 PMC4377062

[11] Loke SR, Andy-Tan WA, Benjamin S, Lee HL, Sofian-Azirun M. Susceptibility of field-collected *Aedes aegypti* (L.) (Diptera: Culicidae) to *Bacillus thuringiensis israelensis* and temephos. Tropical Biomedicine. 2010; 27(3): 493–503. 21399591

[12] Rosilawati R, Lee HL, Nazni WA, Nurulhusna AH, Roziah A, Khairul AM, et al Pyrethroid resistance status of *Aedes* (Stegomyia) *aegypti* (Linneaus) from dengue endemic areas in Peninsular Malaysia. International Medical Journal Malaysia. 2017; 16 (2): 1–6.

[13] Matusop A, Chong JH, Lubim D, Sundin NH. Dengue control: are we dealing with resistant *Aedes* mosquitoes?. Poster presented at the 7th Sarawak Health Department Research Day, 29–30 September, 2015, Sarawak, Malaysia. Abstract published in the program book.

[14] Lau KW, Chen CD, Azidah AA, Sofian-Azirun M. Insecticide resistance detection on dengue vector, *Aedes albopictus* obtained from Kapit, Kuching and Sibu districts in Sarawak State, Malaysia. World Academy of Science, Engineering and Technology International Journal of Bioengineering and Life Sciences. 2015; 9 (11).

[15] Laws of Malaysia. Act 149. Pesticides Act 1974. Updated as at 1 June 2015.

[16] Ministry of Health, Malaysia. National dengue strategic plan for dengue prevention and control in Malaysia, 2009–2013.

[17] Sistem eDengue v2. Sistem pengurusan dan pemantauan aktiviti denggi yang berkesan di seluruh negara secara atas talian dan real time. //edenguev2.moh.gov.my.

[18] Laws of Malaysia ACT 154 Destruction of Disease-Bearing Insects Act 1975. www.moh.gov.my/index.php/database_stores/attach_download/317/7

[19] Lee HL. *Aedes* ovitrap and larval survey in several suburban communities in Selangor, Malaysia. Mosquito Borne Diseases Bulletin. 1992; 9(1): 9–15.

[20] Institute for Public Health. National health and morbidity survey 2011. Volume 1: Methodology and General Findings, 258 pages. The corresponding page is 44.

[21] Benjamin S, Lee HL. Field trials to determine the effectiveness of *Bacillus thuringiensis* subsp. *israelensis* application using an ultra- low-volume generator for the control of *Aedes* mosquitoes. Israel Journal of Entomology. 1998; 32: 25–31.

[22] Benjamin S, Lee HL, Chiang YF. Compatibility of *Bacillus thuringiensis israelensis* and chemical insecticides for the control of *Aedes* mosquitoes. Journal Vector Ecology. 1999; 24: 216–23. 10672551

[23] Benjamin S, Lee HL, Chiang YF. Thermal application of *Bacillus thuringiensis* serovar *israelensis* for dengue vector control. Journal Vector Ecology. 2001; 110–112. 10.1890/0012-9658(2001)082[2673:spdale]2.0.co;2 11469179

[24] Centers for Disease Control and Prevention (CDC). Mosquito-borne transmission. Dengue virus and dengue. https://www.cdc.gov/dengue/training

[25] Vezzani D, Schweigmann N. Suitability of containers from different sources as breeding sites of *Aedes aegypti* (L.) in a cemetery of Buenos Aires City, Argentina. Memórias Instituto Oswaldo Cruz, Rio de Janeiro. 2002; 97(6), 789–792. 10.1590/s0074-02762002000600006 12386697

[26] Jacups SP, Rapley LP, Johnson PH, Benjamin S, Ritchie SA. *Bacillus thuringiensis* var. *israelensis* misting for control of *Aedes* in cryptic ground containers in North Queensland, Australia. The American Journal of Tropical Medicine and Hygiene. 2013; 88:490–6. 10.4269/ajtmh.12-0385 23358637 PMC3592530

[27] Sun D, Williges E, Unlu I, Healy S, Williams GM, Obenauer P et al Taming a tiger in the city: comparison of motorized backpack applications and source reduction against the Asian tiger mosquito, *Aedes albopictus*. Journal of the American Mosquito Control Association. 2014; 30(2):99–105. 10.2987/13-6394.1 25102592

[28] Pruszynski CA, Haribar LJ, Mickle R, Leal AL. A large scale biorational approach using *Bacillus thuringiensis israelensis* (Strain AM65-52) for managing *Aedes aegypti* populations to prevent Dengue, Chikungunya and Zika transmission. PLoS ONE. 2017; 12(2): e0170079 10.1371/journal.pone.0170079 28199323 PMC5310849

[29] Tan AWA, Loke SR, Benjamin S, Lee HL, Chooi KH, Sofian-Azirun M. Spray application of *Bacillus thuringiensis israelensis* (BTI strain AM 65–52) against *Aedes aegypti* (L.) and *Aedes albopictus* (Skuse) populations and impact on dengue transmission in a dengue endemic residential site in Malaysia. Southeast Asian Journal Tropical Medicine and Public Health. 2012; 43:296–310. 23082582

[30] Stoddard PK. Managing *Aedes aegypti* populations in the first Zika transmission zones in the continental United States. Acta Tropica. 2018; 187: 108–118. 10.1016/j.actatropica.2018.07.031 30075097

[31] Likos A, Griffin I, Bingham AM, Stanek D, Fischer M, White S, et al Local mosquito-borne transmission of Zika virus—Miami-Dade and Broward Counties, Florida, June–August 2016. Centers for Disease Control and Prevention Weekly/ September 30, 2016 / 65(38); 1032–1038. http://www.cdc.gov/mmwr/volumes/65/wr/mm6538e1.htm. 10.15585/mmwr.mm6538e1 27684886

[32] Miami-Dade County Solid Waste Management. Mosquito control as updated on 12 July 2017. http://www.miamidade.gov/solidwaster/aerial-spraying.asp.

[33] Azami Muhammad et al Dengue epidemic in Malaysia: Not a predominantly urban disease anymore. BMC Research Notes. 2011; 4:216 10.1186/1756-0500-4-216 21714858 PMC3154160

[34] Lum LCS, Suaya JA, Tan LH, Sah BK, Shepard DS. Quality of Life of Dengue Patients. American Journal Tropical Medicine and Hygiene. 2008; 78(6): 862–867. 18541760

[35] Shepard DS, Undurraga EA, Lees RS, Halasa Y, Lum LCS, Ng CW. Use of Multiple Data Sources to Estimate the Economic Cost of Dengue Illness in Malaysia. American Journal Tropical Medicine and Hygiene. 2012; 87(5): 796–805. 10.4269/ajtmh.2012.12-0019 PMC3516253 23033404

[36] Packierisamy PR, Ng CW, Dahlui M, Inbaraj J, Balan VK, Halasa YA, et al Cost of dengue vector control activities in Malaysia. American Journal of Tropical Medicine and Hygiene. 2015; 93(5): 1020–1027. 10.4269/ajtmh.14-0667 26416116 PMC4703248

[37] Yap ZHA, Chen CD, Azirun MS, Low VL. Pyrethroid resistance in the dengue vector *Aedes aegypti* in Southeast Asia: present situation and prospects for management. Parasites & Vectors. 2018; 11:332 10.1186/s13071-018-2899-0. 29866193 PMC5987412

[38] Eitan Ben-Dov. *Bacillus thuringiensis* subsp. *israelensis* and its Dipteran-specific toxins. Toxins 2014; 6: 1222–1243. 10.3390/toxins6041222 24686769 PMC4014730

[39] Lee HL, Chen CD, Masri SM, Chiang YF, Chooi KH, Benjamin S. Impact of larviciding with a *Bacillus thuringiensis israelensis* formulation, VectoBac WG, on dengue mosquito vectors in a dengue endemic site in Selangor state, Malaysia. Southeast Asian Journal Tropical Medicine and Public Health. 2008; 39:601–9. 19058596

[40] Lam PHY, Chia SB, Ng YY, Benjamin S. *Aedes albopictus* control with spray application of *Bacillus thuringiensis israelensis*, strain AM 65–52. Southeast Asian Journal Tropical Medicine and Public Health. 2010; 41:1071–81. 21073027

[41] Miami-Dade County. Miami-Dade county solid waste management. 2017. http://www.miamidade.gov/solidwaster/aerial-spraying.asp

