# Supplementary material for: Wide area spray of bacterial larvicide, Bacillus thuringiensis israelensis strain AM65-52, integrated in the national vector control program impacts dengue transmission in an urban township in Sibu district, Sarawak, Malaysia
Source: PLoS One. 2020 Apr 1;15(4):e0230910. doi: 10.1371/journal.pone.0230910 (PMC7112204; doi:10.1371/journal.pone.0230910)
Supplement: S1 Data — (PDF) [file pone.0230910.s001.pdf]

UNIT ENTOMOLOGI & PEST
PEJABAT KESIHATAN BAHAGIAN SIBU
MAKLUMAT KAJIAN ENTOMOLOGI OLEH PASUKAN ENTOMOLOGI PKB SIBU

**Bil.**	**Perkara**			**PRE-TREATMENT**
1	Tarikh	**11-14.5.2015**	**20.5.2015**	**27-28.5.2015**
2	Daerah	SIBU	SIBU	SIBU
3	Lokaliti	**JLN AMOY**	**JLN AMOY**	**JLN AMOY**
4	Bil.premis diperiksa	10	15	13
5	Bil.premis positif	2	4	2
6	Bil.kontainer diperiksa	53	129	111
7	Bil kontainer per premis	5.3	8.6	8.6
8	Bil.Kontainer positif	2	5	2
9	Aedes Index (AI) (%)	20.0	26.7	15.4
10	No.of Aedes larvae	2.0	33.0	17.0
11	No. of pupae	4	6	0
12	Larval Density (LD)	0.04	0.26	0.15
13	Pupae Density (PD)	0.08	0.05	0.00
14	Spesies	***Ae.albopictus, Ae.aegypti***	***Ae.albopictus, Ae.aegypti***	***Ae.albopictus, Ae.aegypti***
15	Jenis tempat pembiakan	baldi (2)	tong cat (3), bekas minuman (1), bekas tin (1)	tempayan (1), tin makanan (1)
16	Catatan	Kesemua larva dan pupa hidup	Kesemua larva dan pupa hidup	Kesemua larva hidup

Disediakan oleh;
AKS REZAL BOHARI, PS(KS) C44

		TREATMENT PHASE		
**5.6.2015**	**10.6.2015**	**18.6.2015**	**22.6.2015**	**29.6.2015**
SIBU	SIBU	SIBU	SIBU	SIBU
**JLN AMOY**	**JLN AMOY**	**JLN AMOY**	**JLN AMOY**	**JLN AMOY**
14	15	15	15	15
0	3	5	0	2
89	128	95	74	99
6.4	8.5	6.3	4.93	6.6
0	6	13	0	2
0.0	20.0	33.3	0.0	13.3
0.0	64.0	55.0	0.0	20.0
0	13	37	0	0
0.00	0.50	0.58	0.00	0.20
0.00	0.10	0.39	0.00	0.00
**NIL**	***Ae.albopictus, Ae.aegypti***	***Ae.albopictus, Ae.aegypti, Culex spp.***	**NIL**	***Ae.aegypti***
NIL	tong cat (4), basin (1), pam tandas (1)	tudung cat (3), tin cat (1), tong cat (4), bekas makanan haiwan (2), bekas plastik (1), kuali (1),baldi (1), peti sejuk (1)	NIL	tong cat (2)
NIL	Kesemua larva dan pupa hidup	Kesemua larva dan pupa hidup	NIL	Kesemua larva hidup

**6.7.2015**	**13.7.2015**	**20.7.2015**	**27.7.2015**	**3.8.2015**	**10.8.2015**
SIBU	SIBU	SIBU	SIBU	SIBU	SIBU
**JLN AMOY**	**JLN AMOY**	**JLN AMOY**	**JLN AMOY**	**JLN AMOY**	**JLN AMOY**
15	14	14	15	14	15
2	1	1	1	2	2
101	86	51	74	82	95
6.7	6.14	3.64	4.93	5.9	6.33
2	1	1	3	3	3
13.3	7.1	7.1	6.7	14.3	13.3
12.0	8.0	3.0	28.0	11.0	12.0
0	3	0	5	9	2
0.12	0.09	0.06	0.38	0.13	0.13
0.00	0.035	0.00	0.07	0.11	0.02
Ae.albopictus	***Ae.albopictus, Culex spp.***	***Ae.albopictus***	***Ae.albopictus, Ae.aegypti***	***Ae.albopictus, Ae.aegypti***	***Ae.albopictus, Ae.aegypti***
tayar (1), tong cat (1)	bekas plastik (1)	tong cat (1)	tong cat (3)	tong cat (2), tempayan (1)	tong cat (3)
Kesemua larva hidup	Kesemua larva hidup	Kesemua larva hidup	Kesemua larva hidup	Kesemua larva hidup	Kesemua larva hidup

					POST TREA
**17.8.2015**	**24.8.2015**	**31.8.2015**	**7.9.2015**	**14.9.2015**	**21.9.2015**
SIBU	SIBU	SIBU	SIBU	SIBU	SIBU
**JLN AMOY**	**JLN AMOY**	**JLN AMOY**	**JLN AMOY**	**JLN AMOY**	**JLN AMOY**
15	14	15	15	15	15
2	1	2	1	1	0
67	112	85	65	62	61
4.5	8	5.7	4.3	4.13	4.1
2	2	2	1	1	0
13.3	7.1	13.3	6.7	6.7	0.0
14.0	7.0	13.0	5.0	9.0	0.0
0	1	7	5	0	0
0.21	0.06	0.15	0.08	0.15	0.00
0.00	0.01	0.08	0.08	0.00	0.00
Ae.albopictus	***Ae.albopictus***	***Ae.albopictus***	***Ae.albopictus***	***Ae.aegypti***	***NIL***
bekas plastik (1), tayar (1)	tong cat (1), tin cat (1)	bekas makanan ayam (1), bekas plastik (1)	tong cat (1)	tong cat (1)	NIL
Kesemua larva hidup	Kesemua larva hidup	Kesemua larva hidup	Kesemua larva hidup	Kesemua larva hidup	NIL

ATMENT PHASE		
**28.9.2015**	**5.10.2015**	**12.10.2015**
SIBU	SIBU	SIBU
**JLN AMOY**	**JLN AMOY**	**JLN AMOY**
15	18	14
1	3	3
69	91	90
4.6	5.1	6.4
1	3	3
6.7	16.7	21.4
9.0	17.0	16.0
0	7	3
0.13	0.19	0.18
0.00	0.08	0.033
Ae.albopictus	***Ae.albopictus***	***Ae.albopictus***
tayar (1)	bekas plastik (1), tong cat (1), bekas plastik minuman haiwan (1)	tong cat (2), bekas minuman ayam (1)
Kesemua larva hidup	Kesemua larva hidup	Kesemua larva hidup
