# Supplementary material for: Wide area spray of bacterial larvicide, Bacillus thuringiensis israelensis strain AM65-52, integrated in the national vector control program impacts dengue transmission in an urban township in Sibu district, Sarawak, Malaysia
Source: PLoS One. 2020 Apr 1;15(4):e0230910. doi: 10.1371/journal.pone.0230910 (PMC7112204; doi:10.1371/journal.pone.0230910)
Supplement: S2 Data — (PDF) [file pone.0230910.s002.pdf]

Field Simulation on the efficacy of Bti Vectobac WG

Objective: To determine the penetration of Bti VectoBac WG in field applications.

Date: 14/04/2015 **Time:** 0800-

Sprayer: Stihl SR 420

Spray Time: 0903-0928 (25 minutes, with orifice 2 & 3 intermittently, On and off throughout the spray)

Product: VectoBac WG

Mix Rate: 125 g in 12 L water (1 caj)

Mosquito: *Aedes aegypti* (F1050) 10 larvae in 200 ml water **Stage:** L3 (hatched 14

72-hours after Spray Operation (4th days after)_17 April 2015

No.	Description (From left to Spray; corner end to main road)	No. Larvae	3-h
			Dead
1	1st house, under the staircase, beside the zinc plate	10	10
2	2nd house, first floor, on the ledge	10	10
3	1st house, in the bushes	10	9
4	2nd house, in the bushes	10	10
5	3rd house (green), under the small table	10	10
6	4th house, outside the house, near to the road	10	10
7	5th house, outside the house, by the side wooden gate	10	10
8	6th house, inside the box at the yard compound	10	10
9	6th house, nearby the window	10	10
10	6th house, in the bushes	10	10
11	7th house, on the staircase	10	10
12	7th house, 1st floor, behind the wall of balcony	10	10
13	8th house (corner lot next to main road), behind the zinc plate	10	9
14	8th house, underneath the wood piece	10	10
15	8th house, on the ground, near to the staircase	10	10
17	8th house, in between the rubbish bins	10	10
18	8th house, in the thick bushes (further in compare to container 16)	10	9
19	8th house, in the thick bushes (further in compare to container 18)	10	10
20	8th house, on the staircase to first floor.	10	10
	**Mean**		**98.**
	**SD**		**3.7463**
	**SE**		**0.8594**

72-hours after Spray Operation (4th days after)_17 April 2015
Control

No.	Description (From left to Spray; corner end to main road)	No. Larvae	3-h
			Dead
1	Control 1	10	0
2	Control 2	10	0
3	Control 3	10	0
	**Mean**		**0.**
	**SD**		[illegible]

	**Field Simu**
	**Objective:**
1000	**Date:** 14/0
	**Sprayer:** St
	**Spray Time**
	**Product:** V
	**Mix Rate:** :
/04/2015)	**Mosquito:**
	**Day 7 afte**

**Mortality**				
**our**		**24-hr**		**No.**
**%**		**Dead**	**%**	
100.00		10	100.00	1
100.00		10	100.00	2
90.00		10	100.00	3
100.00		10	100.00	4
100.00		10	100.00	5
100.00		10	100.00	6
100.00		10	100.00	7
100.00		10	100.00	8
100.00		10	100.00	9
100.00		10	100.00	10
100.00		10	100.00	11
100.00		10	100.00	12
90.00		10	100.00	13
100.00		10	100.00	14
100.00		10	100.00	15
100.00		10	100.00	17
90.00		10	100.00	18
100.00		10	100.00	19
100.00		10	100.00	20
**.42**		**100.00**		
**43246**		**0.00**		
**70085**		**0.00**		

Day 7 afte
Control

**Mortality**				
**our**		**24-hr**		**No.**
**%**		**Dead**	**%**	
0.00		0	0.00	1
0.00		0	0.00	2
0.00		0	0.00	3
**00**		**0.00**		
**)**		**0.00**		

lation on the efficacy of Bti Vectobac WG

To determine the penetration of Bti VectoBac WG in field applications.

ı4/2015 **Time:** 0800-1000

:ihl SR 420

: 0903-0928 (25 minutes, with orifice 2 & 3 intermittently, On and off throughout the spray)

ectoBac WG

125 g in 12 L water (1 caj)

Aedes aegypti (F1050) 10 larvae in remaining water **Stage:** L3 (hatched 17/04/2015)

r Spray Operation_20th April 2015

**Description (From left to Spray; corner end to main road)**	**No. Larvae**	**Mort**	
		**5-hour**	
		**Dead**	**%**
1st house, under the staircase, beside the zinc plate	10	10	100.00
2nd house, first floor, on the ledge	10	10	100.00
1st house, in the bushes	10	10	100.00
2nd house, in the bushes	10	10	100.00
3rd house (green), under the small table	10	10	100.00
4th house, outside the house, near to the road	10	10	100.00
5th house, outside the house, by the side wooden gate	10	10	100.00
6th house, inside the box at the yard compound	10	10	100.00
6th house, nearby the window	10	10	100.00
6th house, in the bushes	10	10	100.00
7th house, on the staircase	10	10	100.00
7th house, 1st floor, behind the wall of balcony	10	10	100.00
8th house (corner lot next to main road), behind the zinc plate	10	10	100.00
8th house, underneath the wood piece	10	10	100.00
8th house, on the ground, near to the staircase	10	10	100.00
8th house, in between the rubbish bins	10	10	100.00
8th house, in the thick bushes (further in compare to container 16)	10	10	100.00
8th house, in the thick bushes (further in compare to container 18)	10	10	100.00
8th house, on the staircase to first floor.	10	10	100.00
**Mean**		**100.00**	
**SD**		**0**	
**SE**		**0**	

r Spray Operation_20th April 2015

**Description (From left to Spray; corner end to main road)**	**No. Larvae**	**Mort**	
		**5-hour**	
		**Dead**	**%**
Control 1	10	0	0.00
Control 2	10	0	0.00
Control 3	10	0	0.00
**Mean**		**0.00**	
**SD**		**0**	

Field Simulation on the efficacy of Bti Vectobac WG

Objective: To determine the penetration of Bti VectoBac WG in field applications.

Date: 14/04/2015

Sprayer: Stihl SR 420

Spray Time: 0903-0928 (25 minutes, with orifice 2 & 3 intermittently, On and off thı

Product: VectoBac WG

Mix Rate: 125 g in 12 L water (1 caj)

Mosquito: *Aedes aegypti* (F1050) 10 larvae in remaining water

Day 14 after Spray Operation_27th April 2015

:ality		No.	Description (From left to Spray; corner end to main road)
24-hr			
Dead	%		
10	100.00	1	1st house, under the staircase, beside the zinc plate
10	100.00	2	2nd house, first floor, on the ledge
10	100.00	3	1st house, in the bushes
10	100.00	4	2nd house, in the bushes
10	100.00	5	3rd house (green), under the small table
10	100.00	6	4th house, outside the house, near to the road
10	100.00	7	5th house, outside the house, by the side wooden gate
10	100.00	8	6th house, inside the box at the yard compound
10	100.00	9	6th house, nearby the window
10	100.00	10	6th house, in the bushes
10	100.00	11	7th house, on the staircase
10	100.00	12	7th house, 1st floor, behind the wall of balcony
10	100.00	13	8th house (corner lot next to main road), behind the zinc plate
10	100.00	14	8th house, underneath the wood piece
10	100.00	15	8th house, on the ground, near to the staircase
10	100.00	17	8th house, in between the rubbish bins
10	100.00	18	8th house, in the thick bushes (further in compare to container 16)
10	100.00	19	8th house, in the thick bushes (further in compare to container 18)
10	100.00	20	8th house, on the staircase to first floor.
**100.00**			**Mean**
**0.00**			**SD**
**0.00**			**SE**

Day 14 after Spray Operation_27th April 2015
Control

:ality		No.	Description (From left to Spray; corner end to main road)
24-hr			
Dead	%		
0	0.00	1	Control 1
0	0.00	2	Control 2
0	0.00	3	Control 3
**0.00**			**Mean**
**0**			**SD**

Field Sim

Objectiv

Time: 0800-1000 **Date:** 14

Sprayer:

roughout the spray) **Spray Ti**

Product:

Mix Rate

Stage: L3 (hatched 24/04/2015) **Mosquit**

Day 21 a

No. Larvae	Mortality				No.
	5-hour		24-hr		
	Dead	%	Dead	%	
10	10	100.00	10	100.00	1
10	10	100.00	10	100.00	2
10	10	100.00	10	100.00	3
10	7	70.00	10	100.00	4
10	10	100.00	10	100.00	5
10	10	100.00	10	100.00	6
10	10	100.00	10	100.00	7
10	10	100.00	10	100.00	8
10	9	90.00	10	100.00	9
10	10	100.00	10	100.00	10
10	9	90.00	10	100.00	11
10	10	100.00	10	100.00	12
10	10	100.00	10	100.00	13
10	10	100.00	10	100.00	14
10	10	100.00	10	100.00	15
10	10	100.00	10	100.00	17
10	10	100.00	10	100.00	18
10	10	100.00	10	100.00	19
10	10	100.00	10	100.00	20
	**97.37**		**100.00**		
	**7.334928056**		**0.00**		
	**1.682747903**		**0.00**		

Day 21 a

Control

No. Larvae	Mortality				No.
	5-hour		24-hr		
	Dead	%	Dead	%	
10	0	0.00	0	0.00	1
10	0	0.00	1	10.00	2
10	0	0.00	0	0.00	3
	**0.00**		**3.33**		
	**0**		**5.77**		

ulation on the efficacy of Bti Vectobac WG

e: To determine the penetration of Bti VectoBac WG in field applications.

/04/2015 **Time:** 0800-1000

Stihl SR 420

me: 0903-0928 (25 minutes, with orifice 2 & 3 intermittently, On and off throughout the spray)

: VectoBac WG

e: 125 g in 12 L water (1 caj)

o: *Aedes aegypti* (F1050) 10 larvae in remaining water **Stage:** L3 (hatched 01/05/2015

fter Spray Operation_04 May 2015

Description (From left to Spray; corner end to main road)	No. Larvae	Mor	
		3-hour	
		Dead	%
1st house, under the staircase, beside the zinc plate	10	10	100.00
2nd house, first floor, on the ledge	10	10	100.00
1st house, in the bushes	10	10	100.00
2nd house, in the bushes	10	7	70.00
3rd house (green), under the small table	10	10	100.00
4th house, outside the house, near to the road	10	10	100.00
5th house, outside the house, by the side wooden gate	10	10	100.00
6th house, inside the box at the yard compound	10	10	100.00
6th house, nearby the window	10	9	90.00
6th house, in the bushes	10	10	100.00
7th house, on the staircase	10	9	90.00
7th house, 1st floor, behind the wall of balcony	10	10	100.00
8th house (corner lot next to main road), behind the zinc plate	10	10	100.00
8th house, underneath the wood piece	10	10	100.00
8th house, on the ground, near to the staircase	10	10	100.00
8th house, in between the rubbish bins	10	10	100.00
8th house, in the thick bushes (further in compare to container 16)	10	10	100.00
8th house, in the thick bushes (further in compare to container 18)	10	10	100.00
8th house, on the staircase to first floor.	10	10	100.00
**Mean**		**97.37**	
**SD**		**7.334928056**	
**SE**		**1.682747903**	

fter Spray Operation_04 May 2015

Description (From left to Spray; corner end to main road)	No. Larvae	Mor	
		3-hour	
		Dead	%
Control 1	10	0	0.00
Control 2	10	0	0.00
Control 3	10	0	0.00
**Mean**		**0.00**	
**SD**		**0**	

Field Simulation on the efficacy of Bti Vectobac WG

Objective: To determine the penetration of Bti VectoBac WG in field applications.

Date: 14/04/2015

Sprayer: Stihl SR 420

Spray Time: 0903-0928 (25 minutes, with orifice 2 & 3 intermittently, On and off thı

Product: VectoBac WG

Mix Rate: 125 g in 12 L water (1 caj)

5) **Mosquito:** *Aedes aegypti* (F1052) 10 larvae/cup in remaining water + refill another

Day 28 after Spray Operation_11 May 2015

·tality		No.	Description (From left to Spray; corner end to main road)
24-hr			
Dead	%		
10	100.00	1	1st house, under the staircase, beside the zinc plate
10	100.00	2	2nd house, first floor, on the ledge
10	100.00	3	1st house, in the bushes
10	100.00	4	2nd house, in the bushes
10	100.00	5	3rd house (green), under the small table
10	100.00	6	4th house, outside the house, near to the road
10	100.00	7	5th house, outside the house, by the side wooden gate
10	100.00	8	6th house, inside the box at the yard compound
10	100.00	9	6th house, nearby the window
10	100.00	10	6th house, in the bushes
10	100.00	11	7th house, on the staircase
10	100.00	12	7th house, 1st floor, behind the wall of balcony
10	100.00	13	8th house (corner lot next to main road), behind the zinc plate
10	100.00	14	8th house, underneath the wood piece
10	100.00	15	8th house, on the ground, near to the staircase
10	100.00	17	8th house, in between the rubbish bins
10	100.00	18	8th house, in the thick bushes (further in compare to container 16)
10	100.00	19	8th house, in the thick bushes (further in compare to container 18)
10	100.00	20	8th house, on the staircase to first floor.
**100.00**			**Mean**
**0.00**			**SD**
**0.00**			**SE**

Day 21 after Spray Operation_04 May 2015
Control

·tality		No.	Description (From left to Spray; corner end to main road)
24-hr			
Dead	%		
0	0.00	1	Control 1
0	0.00	2	Control 2
0	0.00	3	Control 3
**0.00**			**Mean**
**0.00**			**SD**

Time: 0800-1000

roughout the spray)

200 ml water

Stage: L3 (hatched 08/05/2015, 5pm)

No. Larvae	Mortality								
	3-hour		24-hr		48-Hr		72-Hr		96-
	Dead	%	Dead	%	Dead	%	Dead	%	Dead
10	9	90.00	10	100.00	10	100.00	10	100.00	10
10	1	10.00	9	90.00	10	100.00	10	100.00	10
10	1	10.00	9	90.00	9	90.00	10	100.00	10
10	6	60.00	10	100.00	10	100.00	10	100.00	10
10	1	10.00	8	80.00	10	100.00	10	100.00	10
10	3	30.00	10	100.00	10	100.00	10	100.00	10
10	1	10.00	10	100.00	10	100.00	10	100.00	10
10	3	30.00	10	100.00	10	100.00	10	100.00	10
10	10	100.00	10	100.00	10	100.00	10	100.00	10
10	2	20.00	10	100.00	10	100.00	10	100.00	10
10	10	100.00	10	100.00	10	100.00	10	100.00	10
10	3	30.00	10	100.00	10	100.00	10	100.00	10
10	9	90.00	10	100.00	10	100.00	10	100.00	10
10	0	0.00	10	100.00	10	100.00	10	100.00	10
10	2	20.00	10	100.00	10	100.00	10	100.00	10
10	0	0.00	9	90.00	10	100.00	10	100.00	10
10	1	10.00	10	100.00	10	100.00	10	100.00	10
10	0	0.00	8	80.00	8	80.00	9	90.00	10
10	10	100.00	10	100.00	10	100.00	10	100.00	10
	**37.89**		**96.32**		**98.42**		**99.47**		**100**
	**38.38**		**6.84**		**5.01**		**2.29**		**0.[illegible]**
	**8.81**		**1.57**		**1.15**		**0.53**		**0.[illegible]**

Day 28 after Spray Operation_11 May 2015
Control

No. Larvae	Mortality				No.	Descriptio	No. Larvae		
	3-hour		24-hr					3-hour	
	Dead	%	Dead	%				Dead	%
10	0	0.00	0	0.00	1	Control 1	10	0	0.00
10	0	0.00	0	0.00	2	Control 2	10	0	0.00
10	0	0.00	0	0.00	3	Control 3	10	0	0.00
	**0.00**		**0.00**			**Mean**		**0.00**	
	**0**		**0.00**			**SD**		**0**	

-Hr
%
100.00
100.00
100.00
100.00
100.00
100.00
100.00
100.00
100.00
100.00
100.00
100.00
100.00
100.00
100.00
100.00
100.00
100.00
100.00
**.00**
**00**
**00**

Mortality							
**24-hr**		**48-hr**		**72-hr**		**72-hr**	
**Dead**	**%**	**Dead**	**%**	**Dead**	**%**	**Dead**	**%**
0	0.00	0	0.00	0	0.00	0	0.00
0	0.00	0	0.00	0	0.00	1	10.00
0	0.00	0	0.00	0	0.00	0	0.00
**0.00**		**0.00**		**0.00**		**3.33**	
**0.00**		**0.00**		**0.00**		**5.77**	
