# Supplementary material for: Wide area spray of bacterial larvicide, Bacillus thuringiensis israelensis strain AM65-52, integrated in the national vector control program impacts dengue transmission in an urban township in Sibu district, Sarawak, Malaysia
Source: PLoS One. 2020 Apr 1;15(4):e0230910. doi: 10.1371/journal.pone.0230910 (PMC7112204; doi:10.1371/journal.pone.0230910)
Supplement: S3 Data — (PDF) [file pone.0230910.s003.pdf]

Ovitrap Index

30 ovitraps placed per site

		Ovitrap Index	
	**EW**	**Untreated site Z-5**	**Bti treated site Z-7L**
	19	10.0	16.7
	21	0.0	3.7
	23	10.0	6.7
Treatment Phase	*25*	13.3	3.6
Treatment Phase	*27*	10.0	10.0
Treatment Phase	*31*	22.2	14.8
Treatment Phase	*33*	33.3	11.5
Treatment Phase	*35*	26.7	3.3
	37	56.7	23.3
	39	43.3	10.0
	41	36.7	25.9
